# Modeling autosomal recessive cutis laxa type 1C in mice reveals distinct functions for Ltbp-4 isoforms

**DOI:** 10.1242/dmm.018960

**Published:** 2015-02-20

**Authors:** Insa Bultmann-Mellin, Anne Conradi, Alexandra C. Maul, Katharina Dinger, Frank Wempe, Alexander P. Wohl, Thomas Imhof, F. Thomas Wunderlich, Alexander C. Bunck, Tomoyuki Nakamura, Katri Koli, Wilhelm Bloch, Alexander Ghanem, Andrea Heinz, Harald von Melchner, Gerhard Sengle, Anja Sterner-Kock

**Affiliations:** 1 Center for Experimental Medicine, Medical Faculty, University of Cologne, 50931 Cologne, Germany.; 2 Department of Pediatrics and Adolescent Medicine, Medical Faculty, University of Cologne, 50937 Cologne, Germany.; 3 Department of Molecular Hematology, University of Frankfurt Medical School, 60590 Frankfurt am Main, Germany.; 4 Center for Biochemistry, Medical Faculty, University of Cologne, 50931 Cologne, Germany.; 5 Center for Molecular Medicine Cologne (CMMC), University of Cologne, 50931 Cologne, Germany.; 6 Institute for Dental Research and Oral Musculoskeletal Biology, Medical Faculty, University of Cologne, 50931 Cologne, Germany.; 7 Max Planck Institute for Metabolism Research, 50931 Cologne, Germany.; 8 Cologne Excellence Cluster on Cellular Stress Responses in Aging-Associated Diseases (CECAD), University of Cologne, 50931 Cologne, Germany.; 9 Department of Radiology, Medical Faculty, University of Cologne, 50937 Cologne, Germany.; 10 Department of Pharmacology, Kansai Medical University, Osaka 570-8506, Japan.; 11 Research Programs Unit and Transplantation Laboratory, Haartman Institute, University of Helsinki, 00014 Helsinki, Finland.; 12 Institute of Cardiology and Sports Medicine, German Sport University Cologne, 50933 Cologne, Germany.; 13 Department of Medicine/Cardiology, University of Bonn, 53127 Bonn, Germany.; 14 Institute of Pharmacy, Martin Luther University Halle-Wittenberg, 06120 Halle (Saale), Germany.

**Keywords:** Latent transforming growth factor β-binding protein 4, Ltbp-4, Ltbp-4L, Ltbp-4S, Autosomal recessive cutis laxa type 1C, ARCL1C, Elastogenesis, Extracellular matrix, ECM, Fibulin-4, Fibulin-5

## Abstract

Recent studies have revealed an important role for LTBP-4 in elastogenesis. Its mutational inactivation in humans causes autosomal recessive cutis laxa type 1C (ARCL1C), which is a severe disorder caused by defects of the elastic fiber network. Although the human gene involved in ARCL1C has been discovered based on similar elastic fiber abnormalities exhibited by mice lacking the short Ltbp-4 isoform (*Ltbp4S*^−/−^), the murine phenotype does not replicate ARCL1C. We therefore inactivated both Ltbp-4 isoforms in the mouse germline to model ARCL1C. Comparative analysis of *Ltbp4S*^−/−^ and *Ltbp4*-null (*Ltbp4*^−/−^) mice identified Ltbp-4L as an important factor for elastogenesis and postnatal survival, and showed that it has distinct tissue expression patterns and specific molecular functions. We identified fibulin-4 as a previously unknown interaction partner of both Ltbp-4 isoforms and demonstrated that at least Ltbp-4L expression is essential for incorporation of fibulin-4 into the extracellular matrix (ECM). Overall, our results contribute to the current understanding of elastogenesis and provide an animal model of ARCL1C.

## INTRODUCTION

The extracellular matrix (ECM) determines and controls the biochemical and mechanical properties of all mammalian tissues including specific ligand-binding properties, elasticity and stiffness (Frantz et al., 2010). As integral ECM components, elastic fibers enable elastic recoil and resilience in all elastic tissues such as arteries, lung, skin and cartilage. Despite being indispensable for survival and normal tissue function (Hornstra et al., 2003; Li et al., 1998), the molecular mechanisms controlling elastic fiber formation have only recently been investigated in more detail (Choudhury et al., 2009; Dabovic et al., 2015; Hirai et al., 2007; Horiguchi et al., 2009; Noda et al., 2013; Sideek et al., 2014). Elastic fibers consist of an internal core of cross-linked monomeric elastin (tropoelastin) surrounded by a network of fibrillin-rich microfibrils (Kielty et al., 2002; Mithieux and Weiss, 2005). Elastic fiber formation in the ECM is a complex process, which involves the formation of fibrillin-rich microfibrillar scaffolds and the deposition of the tropoelastin molecules onto these scaffolds. These processes are followed by the cross-linking of tropoelastin after oxidative deamination of lysine residues by enzymes of the lysyl oxidase family, leading to the formation of covalent cross-links such as desmosines and isodesmosines (Mithieux and Weiss, 2005; Wagenseil and Mecham, 2007; Yanagisawa and Davis, 2010). This process is strongly influenced by the composition of the microfibrillar scaffold. Although the role of several microfibrillar proteins in elastic fiber formation has been studied in more detail (Mithieux and Weiss, 2005; Ramirez et al., 2004; Todorovic and Rifkin, 2012), the impact of the microfibril-associated latent transforming growth factor β-binding protein 4 (LTBP-4) on elastic fiber formation is still poorly understood.

LTBP-4 belongs to a family of four (LTBP-1–LTBP-4) secreted ECM glycoproteins with structural homology to fibrillins (fibrillin-1–fibrillin-3). LTBP-1, LTBP-3 and LTBP-4 covalently bind the small latent complex (SLC) of TGFβ (TGFβ-LAP; LAP, latency-associated peptide) and deposit these complexes into the ECM after secretion (Todorovic and Rifkin, 2012). LTBP-1 and LTBP-3 bind all three TGFβ isoforms, whereas LTBP-4 binds only TGFβ1 (Saharinen et al., 1998). In addition to a TGFβ-related function, LTBPs have structural functions within the ECM (Isogai et al., 2003). For example, LTBP-2, a non TGFβ carrier, interacts with fibulin-5 and thus negatively regulates elastogenesis (Sideek et al., 2014), whereas LTBP-4 facilitates elastogenesis by interacting with fibulin-5 (Dabovic et al., 2015; Noda et al., 2013).

Mammalian cells express two major isoforms of LTBP-4, which in analogy to the long and short isoforms of LTBP-1 discovered earlier are also called long (LTBP-4L; human: NM_001042544.1; murine: NM_175641.2) and short (LTBP-4S; human, NM_001042545.1; murine, NM_001113549.1). These isoforms are encoded by two N-terminal splice variants that are independently expressed using their own promoters (Kantola et al., 2010). In humans LTBP-4L and LTBP-4S are differentially expressed in a tissue-specific manner and show distinct deposition patterns in the ECM (Kantola et al., 2010), presumably due to their different N-termini. For ECM-targeting, the N-terminus of LTBP-4 appears to be particularly important because it enables binding to fibronectin and thereby guides LTBP-4 deposition into the early ECM (Kantola et al., 2008).

**TRANSLATIONAL IMPACT**

**Clinical issue**

Recent studies revealed an important role for latent TGFβ-binding protein-4 (LTBP-4) in elastogenesis (formation of elastic fibers). Its mutational inactivation in humans causes autosomal recessive cutis laxa type 1C (ARCL1C), which is a severe disorder caused by defects of the elastic fiber network. Although the human gene involved in ARCL1C was discovered based on similar elastic fiber abnormalities exhibited by *Ltbp4S*^−/−^ mice (which lack the short Ltbp-4 isoform), the murine phenotype does not replicate human ARCL1C. The authors therefore hypothesize that inactivation of both the long (Ltbp-4L) and short Ltbp-4 isoforms in the mouse germline might model ARCL1C.

**Results**

Comparative analysis of *Ltbp4S*^−/−^ and Ltbp4-null (*Ltbp4*^−/−^) mice identify important and partially non-overlapping functions of Ltbp-4L and Ltbp-4S in survival and elastic fiber formation, as well as unique and overlapping expression of the two isoforms depending on the analyzed tissue, with characteristic tissue localization patterns. Furthermore, the authors identify fibulin-4 as a new interaction partner of both Ltbp-4 isoforms and demonstrate that Ltbp-4L is crucial for postnatal survival. Moreover, the authors show that specific N-glycosylation of Ltbp-4S modulates its binding affinity to fibulin-4- and fibulin-5.

**Implications and future directions**

Overall, the results contribute to the current understanding of elastogenesis and provide an animal model of ARCL1C. The study documents that Ltbp-4L and Ltbp-4S have distinct roles in elastic fiber formation and postnatal survival in mice and identify fibulin-4 as an interaction partner of both Ltbp-4 isoforms. Future experiments involving the generation of *Ltbp4L*-knockout and tissue-specific-*Ltbp4*-knockout mouse lines are planned to gain further insights into the isoform-specific functions of Ltbp-4.

We and others have previously reported that mice with an inactivating mutation of *Ltbp4S* (*Ltbp4S*^−/−^ mice) are born with alveolar septation defects that deteriorate with age and eventually develop massive pulmonary emphysema associated with dilated cardiomyopathy (Dabovic et al., 2009; Sterner-Kock et al., 2002).

In humans, null mutations in the *LTBP4* gene cause autosomal recessive cutis laxa type 1C (ARCL1C; initially called Urban-Rifkin-Davis syndrome), which is a rare congenital connective tissue disorder characterized by severe craniofacial anomalies, lax skin and severe abnormalities in several visceral organs including the lung. However, ARCL1C patients have several lesions that have not been described in *Ltbp4S*^−/−^ mice (Callewaert et al., 2013; Sterner-Kock et al., 2002; Urban et al., 2009). A possible explanation for this could be that Ltbp-4L expressed in *Ltbp4S*^−/−^ mice compensates in part for the loss of Ltbp-4S.

To address Ltbp-4 isoform-specific functions, we generated *Ltbp4*-null (*Ltbp4*^−/−^) mice that, like the ARCL1C patients, do not express any Ltbp-4. Here, we show that *Ltbp4*^−/−^ mice nearly replicate the features of ARCL1C syndrome. We further show that Ltbp-4L and Ltbp-4S have important and partially non-overlapping roles in survival and elastic fiber formation, identify fibulin-4 as a new interacting partner of both Ltbp-4 isoforms and demonstrate that at least Ltbp-4L expression is essential for incorporation of fibulin-4 into the ECM.

## RESULTS

### Complete inactivation of the *Ltbp4* gene in mice results in weight loss, growth reduction and neonatal death

**Fig. 1.**
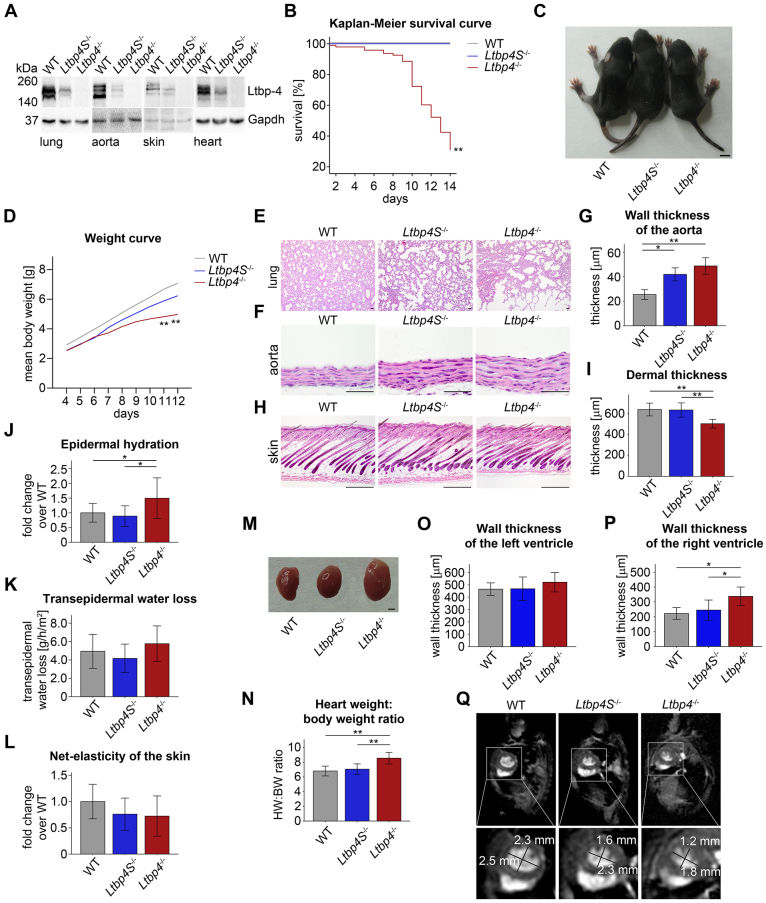
**Clinical and morphological analyses of Ltbp-4 deficient mice.** (A) Representative immunoblots of lung, aorta, skin and hearts showing the reduced or absent expression of Ltbp-4 in *Ltbp4S*^−/−^ and *Ltbp4*^−/−^ mice compared to WT mice. (B) Kaplan–Meier survival curve revealing the significantly higher neonatal mortality in *Ltbp4*^−/−^ mice compared to *Ltbp4S*^−/−^ and WT mice (*n*≥23; **P<0.01 versus WT). (C) *Ltbp4*^−/−^ mice showed reduced body size compared to *Ltbp4S*^−/−^ and WT mice. Scale bar: 0.5 cm. (D) The P4-P12 weight curve showed significantly reduced body weight of *Ltbp4*^−/−^ mice compared to *Ltbp4S*^−/−^ and WT mice (*n*≥8; **P<0.01 versus WT). (E) In *Ltbp4S*^−/−^ mice, the pulmonary parenchyma showed enlarged alveolar spaces with reduced numbers of alveoli and multifocal areas of atelectasis compared to WT mice. *Ltbp4*^−/−^ lungs revealed lack of lobular architecture, severely enlarged alveolar spaces and emphysematous areas compared to WT mice. Scale bars: 40 μm. (F,G) Aortas showed marked thickening of the aortic wall in *Ltbp4S*^−/−^ and *Ltbp4*^−/−^ mice compared to WT mice (*n*≥3; *P<0.05, **P<0.01). Scale bars: 40 μm. (H,I) *Ltbp4*^−/−^ mice showed reduced dermal thickness compared to *Ltbp4S*^−/−^ and WT mice (*n*≥5;**P<0.01). Scale bars: 200 μm. (J) The epidermal hydration was increased in *Ltbp4*^−/−^ mice compared to *Ltbp4S*^−/−^ and WT mice (*n*≥9; *P<0.05). (K) The transepidermal water loss tended to be higher in *Ltbp4*^−/−^ mice compared to *Ltbp4S*^−/−^ and WT mice (*n*≥9; not significant). (L) The net-elasticity of the skin of both Ltbp-4-deficient mice tended to be lower compared to WT mice (*n*≥9; not significant). (M) Representative images showing the increased size of hearts of *Ltbp4*^−/−^ mice compared to hearts of WT and *Ltbp4S*^−/−^ mice. Scale bar: 0.1 cm. (N) Heart weight:body weight ratios showing that hearts of *Ltbp4*^−/−^ mice were significantly heavier than hearts of WT and *Ltbp4S*^−/−^ mice (*n*≥7; **P<0.01). (O) Wall thickness of the left ventricle was not changed in *Ltbp4S*^−/−^ and *Ltbp4*^−/−^ mice compared to WT mice (*n*≥6; not significant). (P) Wall thickness of the right ventricle was significantly increased in *Ltbp4*^−/−^ mice compared to *Ltbp4S*^−/−^ and WT mice (*n*≥6; *P<0.05). (Q) Representative short-axis images from MRI analysis revealing a flattened interventricular septum resulting in a more oval shape of the left ventricle in *Ltbp4S*^−/−^ and *Ltbp4*^−/−^ mice compared to WT mice.

We previously established and characterized *Ltbp4S*-knockout (*Ltbp4S*^−/−^) mice (Sterner-Kock et al., 2002), which show similar elastic fiber defects in the lung to those found in ARCL1C patients; however the mice survived to adulthood without manifestation of major clinical symptoms. To investigate whether the less-severe phenotype of *Ltbp4S*^−/−^ mice was due to the presence of Ltbp-4L, which might have important functions during development, we generated *Ltbp4*-null (*Ltbp4*^−/−^) mice lacking both Ltbp-4 isoforms (supplementary material Fig. S1). Indeed, unlike *Ltbp4S*^−/−^ mice, *Ltbp4*^−/−^ mice showed no expression of Ltbp-4 (Fig. 1A; supplementary material Fig. S2).

Complete lack of Ltbp-4 expression reduced survival dramatically as most *Ltbp4*^−/−^mice died between P8 and P14 (Fig. 1B), whereas *Ltbp4S*^−/−^ mice survived to adulthood (Sterner-Kock et al., 2002). Furthermore, *Ltbp4*^−/−^ mice were smaller than *Ltbp4S*^−/−^ mice and showed decreased body weight as compared with their wild-type (WT) littermates (Fig. 1C,D; Table 1).

**Table 1.**
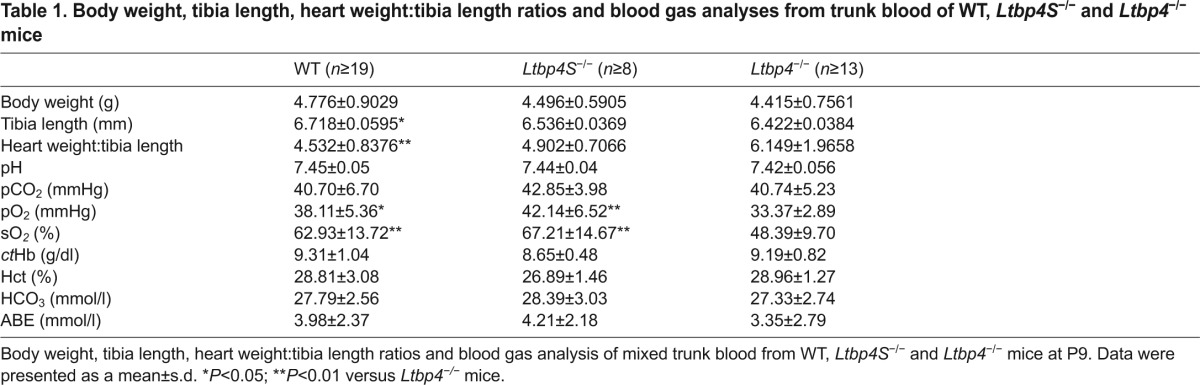
Body weight, tibia length, heart weight:tibia length ratios and blood gas analyses from trunk blood of WT, *Ltbp4S*^−/−^ and *Ltbp4*^−/−^ mice

### Ltbp-4-deficient mice show morphological abnormalities in lung, aorta and skin

Similar to the ARCL1C lung histopathology, the lungs of *Ltbp4*^−/−^ mice lacked a lobular architecture and showed severely enlarged alveolar spaces (Fig. 1E). Lungs from *Ltbp4S*^−/−^ mice also exhibited enlarged alveolar spaces with multifocal areas of atelectasis although these were much less pronounced than in the *Ltbp4*^−/−^ lungs (Fig. 1E). This difference was reflected by a significantly reduced oxygen saturation (sO_2_) and partial oxygen pressure (pO_2_) in *Ltbp4*^−/−^ mice when compared with *Ltbp4S*^−/−^ and WT mice (Table 1).

Aortas of Ltbp-4-deficient mice were malformed and tortuous (supplementary material Fig. S3) (Noda et al., 2013). Aortic walls were twice as thick in *Ltbp4*^−/−^ mice as in WT mice (Fig. 1F,G). The aortic wall thickening could not be attributed to extra elastic lamellae (Fig. 1F), increased deposition of glycosaminoglycans in the ECM, increased numbers of smooth muscle cells or increased proliferation (supplementary material Fig. S4).

*Ltbp4*^−/−^ mice showed reduced dermal thickness compared to *Ltbp4S*^−/−^ and WT mice (Fig. 1H,I), whereas the thickness of the epidermis was not changed (data not shown). The epidermal hydration was increased (Fig. 1J) and the transepidermal water loss (Fig. 1K) tended to be higher but did not reach statistical significance in *Ltbp4*^−/−^ mice compared to *Ltbp4S*^−/−^ and WT mice. The net-elasticity of the skin of both Ltbp-4-deficient mice tended to be lower but did not reach statistical significance compared to WT mice (Fig. 1L).

### *Ltbp4*^−/−^ mice develop cardiac hypertrophy of the right ventricle with flattening of the interventricular septum

Hearts of *Ltbp4*^−/−^ but not of *Ltbp4S*^−/−^ mice were larger when compared to WT hearts (Fig. 1M) as indicated by higher heart to body weight (Fig. 1N) and heart weight to tibia length ratios (Table 1). Stereological analysis revealed significantly increased right ventricular wall thickness in *Ltbp4*^−/−^ hearts as compared to *Ltbp4S*^−/−^ or WT hearts, but showed no change in the left ventricular walls (Fig. 1O,P). The left ventricular end-diastolic volume (LVEDV), the left ventricular end-systolic volume (LVESV) and the stroke volume (SV) tended to be lower and the ejection fraction (EF) tended to be higher but did not reach statistical significance in *Ltbp4*^−/−^ mice compared to WT mice (supplementary material Table S1).

*Ltbp4*^−/−^ mice and to a lesser extent *Ltbp4S*^−/−^ mice develop flattened interventricular septae resulting in a more oval shape of the left ventricle on short axis views (Fig. 1Q). The ratio between the maximum and minimum diameter of the left ventricle measured on end-diastolic short axis images was 1.5 in *Ltbp4*^−/−^ and 1.4 in *Ltbp4S*^−/−^ mice. In contrast, the ratio for WT ventricles was 1.1, consistent with a round ventricle shape (Fig. 1Q).

### Overlapping and distinct expression and localization patterns of Ltbp-4L and Ltbp-4S

Because the different phenotypes of *Ltbp4S*^−/−^ and *Ltbp4*^−/−^ mice might reflect differences in tissue-specific expression and localization of the Ltbp-4 isoforms, we quantified the amount of *Ltbp4L* and *Ltbp4S* transcripts in various tissues of WT, *Ltbp4S*^−/−^ and *Ltbp4*^−/−^ mice and related these results to Ltbp-4 protein expression.

**Fig. 2.**
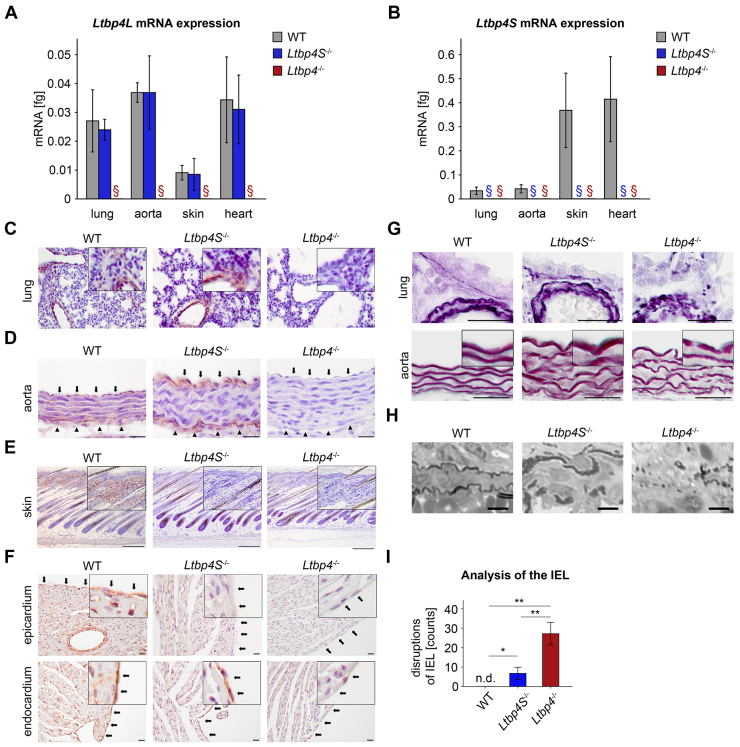
**Localization patterns of Ltbp-4L, Ltbp-4S and elastin.** (A) Quantitative PCRs of lung, aorta, skin and heart of WT and *Ltbp4S*^−/−^ mice showing that each tissue had different amounts of *Ltbp4L* mRNA. There was no mRNA expression of *Ltbp4L* in lung, aorta, skin and heart of *Ltbp4*^−/−^ mice (*n*≥3; §, no expression detectable). (B) Quantitative PCRs of lung, aorta, skin and heart of WT mice showing that each tissue had different amounts of *Ltbp4S* mRNA. There was no mRNA expression of *Ltbp4S* in lung, aorta, skin and heart of *Ltbp4S*^−/−^ and *Ltbp4*^−/−^ mice (*n*≥3; §, no expression detectable). (C) Representative images of Ltbp-4 immunoreactivity of lungs from WT, *Ltbp4S*^−/−^ and *Ltbp4*^−/−^ mice. Ltbp-4 was localized particularly in bronchial and bronchiolar walls and in vascular walls of WT and *Ltbp4S*^−/−^ mice. Lungs of *Ltbp4*^−/−^ mice were negative for Ltbp-4 immunoreactivity. Scale bars: 20 μm. (D) Representative images of Ltbp-4 immunoreactivity of aortas from WT, *Ltbp4S*^−/−^ and *Ltbp4*^−/−^ mice. Black arrows point to the aortic luminal side and black arrowheads to the adventitia. Ltbp-4 immunoreactivity was present in the vicinity of aortic elastic lamella throughout the entire aorta (from the endothelial lining to the adventitia) of WT mice and in the vicinity of the internal elastic lamella (IEL) and in the adventitia of *Ltbp4S*^−/−^ mice. The aortic intramural elastic lamella of *Ltbp4S*^−/−^ mice and the entire aorta of *Ltbp4*^−/−^ mice showed no immunoreactivity for Ltbp-4. Scale bars: 20 μm. (E) Representative images of Ltbp-4 immunoreactivity of skin from WT, *Ltbp4S*^−/−^ and *Ltbp4*^−/−^ mice. In WT skin, Ltbp-4 immunoreactivity was present in the entire dermis, whereas it was completely absent in the epidermis. There was no difference in the tissue distribution of Ltbp-4 between WT and *Ltbp4S*^−/−^ skin. The skin of *Ltbp4*^−/−^ mice expressed no Ltbp-4. Scale bars: 20 μm. (F) Representative images of Ltbp-4 immunoreactivity of hearts from WT, *Ltbp4S*^−/−^ and *Ltbp4*^−/−^ mice. Upper panel: black arrows point to the epicardium of the heart. Ltbp-4 immunoreactivity was present within the myocardium and in the epicardium of WT mice. The myocardium and the epicardium of *Ltbp4S*^−/−^ and *Ltbp4*^−/−^ mice were negative for Ltbp-4 immunoreactivity. Lower panel: black arrows point to the endocardium. The endocardium of WT and *Ltbp4S*^−/−^ mice clearly has Ltbp-4 immunoreactivity, whereas *Ltbp4*^−/−^ mice were negative for Ltbp-4 immunoreactivity. Scale bars: 20 μm. (G) Representative histochemical elastica stainings of lungs (upper panels) and aortas (lower panels) displaying moderate elastic fiber fragmentation with intact and disrupted elastic fibers in *Lbp4S*^−/−^ mice compared to WT mice and an increased degree of fragmentation of the elastic fibers in *Ltbp4*^−/−^ mice compared to *Lbp4S*^−/−^ mice. Scale bars: 20 μm. (H) Representative semi-thin sections of lungs showing elastic fibers with fragmented and intact parts in *Lbp4S*^−/−^ mice and total disruption of elastic fibers in *Ltbp4*^−/−^ mice compared to WT mice. Scale bars: 6 μm. (I) Quantitative analysis of disruptions of the IEL showing significantly higher numbers of disruptions in *Ltbp4*^−/−^ mice compared to WT and *Ltbp4S*^−/−^ mice and significantly higher numbers of disruptions in *Ltbp4S*^−/−^ mice compared to WT mice (*n*=6; n.d., not detectable; *P<0.05, **P<0.01).

WT lungs expressed comparable amounts of *Ltbp4L* and *Ltbp4S* mRNA, whereas in lungs from *Ltbp4S*^−/−^ mice only *Ltbp4L* was expressed. In the lungs of *Ltbp4*^−/−^ mice neither *Ltbp4L* nor *Ltbp4S* mRNA was expressed (Fig. 2A,B). Ltbp-4 immunoreactivity was found primarily in the bronchial and bronchiolar walls, as well as in the parenchyma and vascular walls, of WT lungs (Fig. 2C). There was no difference in the tissue distribution of Ltbp-4 between WT and *Ltbp4S*^−/−^ lungs (Fig. 2C), indicating that Ltbp-4 isoform expression is overlapping in the lung. Ltbp-4 immunoreactivity was completely absent in *Ltbp4*^−/−^ lungs (Fig. 2C).

Aortas of WT mice expressed comparable amounts of *Ltbp4L* and *Ltbp4S* mRNA, whereas the aortas of *Ltbp4S*^−/−^ mice expressed exclusively *Ltbp4L* mRNA. Aortas of *Ltbp4*^−/−^ mice expressed neither *Ltbp4* isoform (Fig. 2A,B). In WT aortas, Ltbp-4 immunoreactivity was present in the vicinity of the aortic elastic lamellae throughout the entire aortic wall extending from the endothelial lining to the adventitia (Fig. 2D). However, in *Ltbp4S*^−/−^ mice, Ltbp-4 was only detectable in the vicinity of the internal elastic lamella (IEL) and in the adventitia. Intramural elastic lamellae of *Ltbp4S*^−/−^ mice were negative for Ltbp-4 staining, suggesting that Ltbp-4L expression is restricted to the adventitia and the IEL (Fig. 2D). Aortas of *Ltbp4*^−/−^ mice expressed no Ltbp-4 (Fig. 2D).

In WT skin, *Ltbp4S* was the major isoform expressed, representing about 98% of the total *Ltbp4* transcripts. In *Ltbp4S*^−/−^ skin, only *Ltbp4L* mRNA was expressed and in *Ltbp4*^−/−^ skin neither *Ltbp4L* nor *Ltbp4S* mRNA was expressed (Fig. 2A,B). In WT skin, Ltbp-4 immunoreactivity was present in the entire dermis and was completely absent in the epidermis (Fig. 2E). There was no difference in the tissue distribution of Ltbp-4 between WT and *Ltbp4S*^−/−^ skin (Fig. 2E), indicating that Ltbp-4 isoform expression is overlapping in the skin. The skin of *Ltbp4*^−/−^ mice expressed no Ltbp-4 (Fig. 2E).

*Ltbp4S* was the major isoform expressed in WT hearts, representing about 93% of the total *Ltbp4* transcripts. Hearts of *Ltbp4S*^−/−^ mice expressed only *Ltbp4L* mRNA. *Ltbp4*^−/−^ hearts expressed neither *Ltbp4L* nor *Ltbp4S* mRNA (Fig. 2A,B). In the heart, Ltbp-4 immunoreactivity was detectable in the epicardium, myocardium and endocardium of WT mice (Fig. 2F). In *Ltbp4S*^−/−^ hearts Ltbp-4 immunoreactivity was only detectable in the endocardium, implying that Ltbp-4L expression is restricted to this region. *Ltbp4*^−/−^ hearts showed no Ltbp-4 immunoreactivity (Fig. 2F).

### Ltbp-4 deficiency in mice results in the failure to form an intact elastic fiber network

We and others have shown that *Ltbp4S*^−/−^ mice display severe defects in elastic fiber formation (Dabovic et al., 2015; Noda et al., 2013; Sterner-Kock et al., 2002; Urban et al., 2009). Elastic fiber fragmentation was present in aortas and lungs of both Ltbp-4-deficient mice (Fig. 2G). In *Ltbp4S*^−/−^ mice, elastic fibers, although fragmented, consisted of short intact fibers dispersed between scattered patches of elastin. In contrast, *Ltbp4*^−/−^ tissues showed only scattered patches of elastin (Fig. 2G). These observations were confirmed on ultrastructural images of semi-thin sections (Fig. 2H, supplementary material Fig. S5A).

Major differences in elastic fiber structure between the Ltbp-4-deficient mice were found in the subluminal region of the aorta, where Ltbp-4L is preferentially expressed (Fig. 2D). Whereas *Ltbp4S*^−/−^ aortas exhibited only moderate disruptions of the IEL adjacent to the endothelial lining, disruptions were significantly more severe in the aortas of *Ltbp4*^−/−^ mice (Fig. 2I).

There were no significant differences in tropoelastin mRNA expression (supplementary material Fig. S5B), in the amount of elastin (supplementary material Fig. S5C) or in the ratio of desmosine (DES) and isodesmosine (IDES) to elastin (supplementary material Fig. S5D) between the genotypes, indicating that the elastic fiber defects observed in the Ltbp-4-deficient mice were not due to altered elastin levels or ineffective elastin cross-linking.

### Ltbp-4 modulates fibulin-5 expression and matrix deposition

It has been suggested that Ltbp-4S promotes elastic fiber assembly by facilitating tropoelastin deposition onto microfibrils through direct interactions with fibulin-5 (Dabovic et al., 2015; Noda et al., 2013). There was an 80% reduction of fibulin-5 mRNA levels in *Ltbp4S*^−/−^ and *Ltbp4*^−/−^ lungs compared to WT lungs (supplementary material Fig. S6A). Moreover, the normal fibrillar structure of fibulin-5 was disrupted in the lungs and aortas of both Ltbp-4-deficient mice and replaced by scattered amorphous patches, suggesting that there is defective deposition of fibulin-5 into the ECM (supplementary material Fig. S6B). *Ltbp4S*^−/−^ fibroblasts expressed ~50% and *Ltbp4*^−/−^ ~10% of the normal amount of fibulin-5 mRNA and protein (supplementary material Fig. S6C,D). Moreover, fibulin-5 ECM deposition was defective in both *Ltbp4S*^−/−^ and *Ltbp4*^−/−^ lung fibroblasts and similar to the ECM deposition observed in the corresponding Ltbp-4-deficient tissues (supplementary material Fig. S6B,E).

### Deposition of fibulin-4 on microfibrils requires Ltbp-4L

**Fig. 3.**
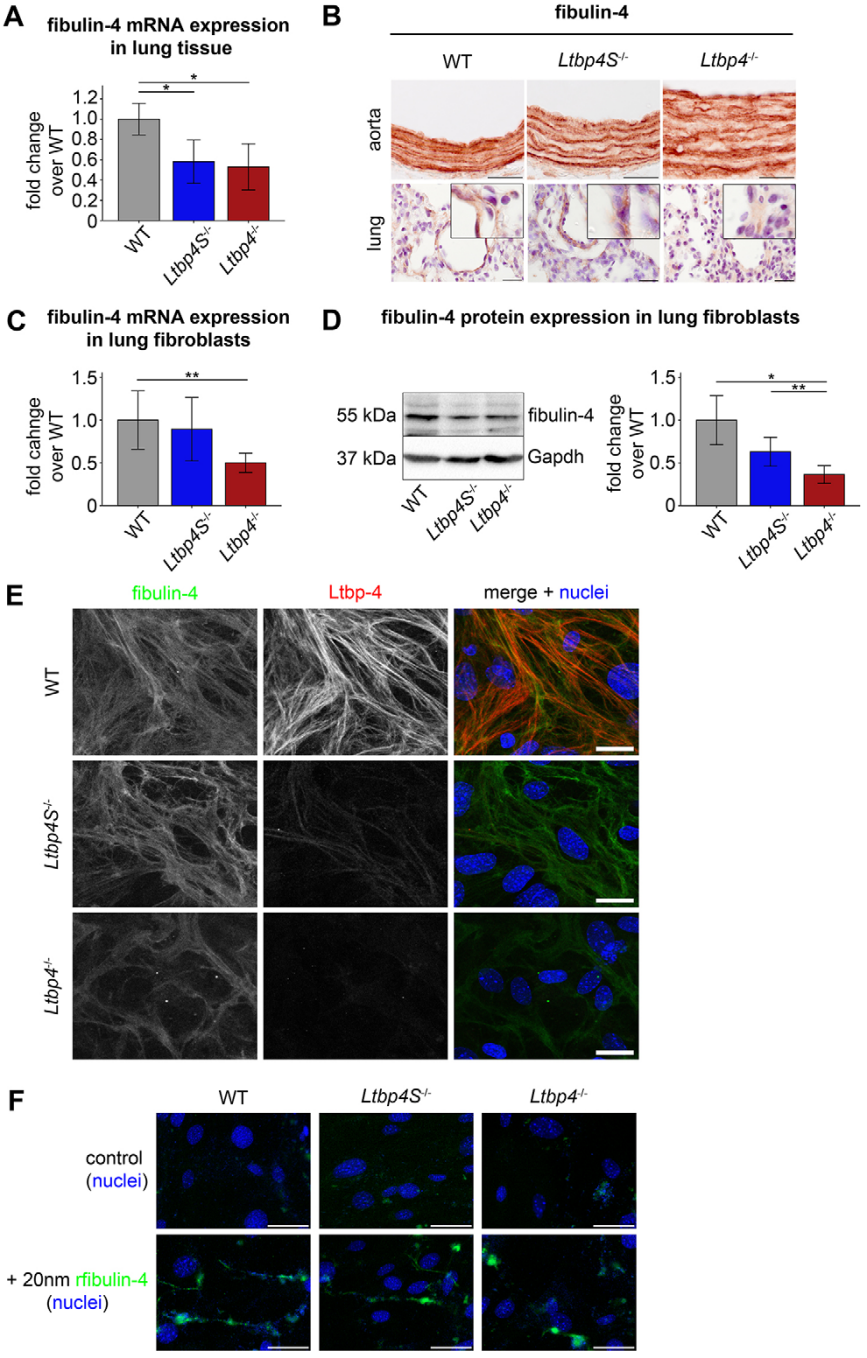
**Ltbp-4L is necessary for fibrillar matrix deposition of fibulin-4.** (A) Fibulin-4 mRNA expression showed significant downregulation in lungs from *Ltbp4S*^−/−^ and *Ltbp4*^−/−^ mice compared to WT mice (*n*=4; *P<0.05). (B) Representative images showing fibulin-4 immunoreactivity and disruption of the fibrillar structure of fibulin-4 fibers in aortas (upper panel) and lungs (lower panel) from *Ltbp4*^−/−^ mice compared to WT and *Ltbp4S*^−/−^ mice. Scale bars: 20 μm (upper panels), 50 μm (lower panels). (C) Fibulin-4 mRNA expression showed significant downregulation in lung fibroblasts isolated from *Ltbp4*^−/−^ mice compared to *Ltbp4S*^−/−^ and WT mice (*n*≥5; **P<0.01). (D) Representative immunoblot of lung fibroblasts (left) and its densitometric analysis (right) revealing significant downregulation of fibulin-4 in *Ltbp4S*^−/−^ and *Ltbp4*^−/−^ mice compared to WT mice (*n*≥4; *P<0.05; **P<0.01). Protein as well as mRNA expression of fibulin-4 of the WT was set to 1. (E) Representative immunofluorescence staining of Ltbp-4 and fibulin-4 revealing reduced expression and disrupted fibrillar structure of fibulin-4 in primary lung fibroblasts from *Ltbp4*^−/−^ mice compared to *Ltbp4S*^−/−^ and WT mice. Scale bars: 100 μm. (F) Representative immunofluorescence staining revealing fibrillar deposition of recombinant full-length fibulin-4 (rfibulin-4; 20 nm) in primary lung fibroblasts from *Ltbp4S*^−/−^ and WT mice, whereas rfibulin-4 appeared scattered and not fibrillar in primary lung fibroblasts from *Ltbp4*^−/−^ mice. For detection of rfibulin-4, an anti-strep antibody was used. Scale bars: 50 μm.

Previous observations in elastin-producing cell cultures have shown that fibulin-4 and fibulin-5 are required for tropoelastin deposition into the ECM (Yamauchi et al., 2010). Although the interaction between Ltbp-4S and fibulin-5 has recently been shown to be essential for elastic fiber formation (Noda et al., 2013), functional interactions between Ltbp-4 and fibulin-4 in this process have not yet been described. The expression of fibulin-4 declined in lungs from both *Ltbp4S*^−/−^ and *Ltbp4*^−/−^ mice (Fig. 3A). However, fibulin-4 deposition differed significantly between the knockout strains. Whereas fibulin-4 deposition into the ECM of *Ltbp4S*^−/−^ mice was comparable to that in WT mice, *Ltbp4*^−/−^ mice exhibited a punctuate deposition pattern (Fig. 3B). We confirmed the decline of fibulin-4 expression in lung fibroblasts from Ltbp-4-deficient mice (Fig. 3C,D), whereas only *Ltbp4*^−/−^ cells showed a defective fibulin-4 matrix deposition (Fig. 3E). Moreover, to normalize fibulin-4 levels, recombinant full-length fibulin-4 (rfibulin-4) was added to primary lung fibroblasts. Primary lung fibroblasts from *Ltbp4S*^−/−^ and WT mice cultured with rfibulin-4 revealed fibrillar deposition of the recombinant protein, whereas the deposition of rfibulin-4 appeared scattered and not fibrillar in primary lung fibroblasts from *Ltbp4*^−/−^ mice (Fig. 3F), suggesting that at least Ltbp-4L is required for fibrillar deposition of fibulin-4.

### N-terminal regions of Ltbp-4L and Ltbp-4S interact with fibulin-4

**Fig. 4.**
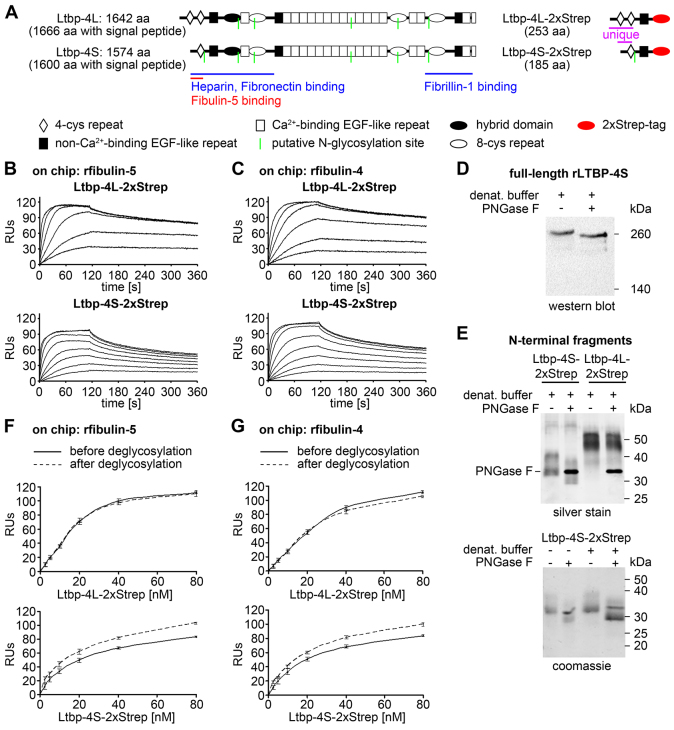
**Interaction studies of the Ltbp-4L and Ltbp-4S N-terminal regions with full-length fibulin-4 and fibulin-5.** (A) Domain structure of full-length Ltbp-4L and Ltbp-4S and the recombinantly expressed Ltbp-4L (Ltbp-4L-2xStrep) and Ltbp-4S (Ltbp-4S-2xStrep) N-terminal fragments. The full-length proteins consist of 4-cystein (4-cys) repeats (white rhombi), non-Ca^2+^-binding EGF-like repeats (black rectangles), Ca^2+^-binding EGF-like repeats (white rectangles), hybrid domains (black ellipses) and 8-cystein (8-cys) repeats (white ellipses). The N-terminal fragments consist of two (Ltbp-4L-2xStrep) or one (Ltbp-4S-2xStrep) unique 4-cys repeats, the common non-Ca^2+^-binding EGF-like repeat and a C-terminal 2xStrep tag (red ellipses). Binding sites for ECM proteins, putative N-glycosylation sites (green lines) as well as the amino acid (aa) lengths are indicated. (B,C) Sensorgrams from surface-plasmon resonance interaction experiments showed a stronger binding affinity of Ltbp-4L-2xStrep (0–320 nM) ‘flown’ over immobilized recombinant full-length fibulin-5 (B; rfibulin-5) or immobilized recombinant full-length fibulin-4 (C; rfibulin-4) compared to Ltbp-4S-2xStrep (0–80 nM) flown over immobilized rfibulin-5 (B) or rfibulin-4 (C). The results are expressed as resonance units (RUs; *n*=2). (D) Deglycosylation digest with PNGase F of denatured recombinant full-length human LTBP-4S (rLTBP-4S) showing that there is a shift towards lower molecular mass positions. (E) Upper panel: deglycosylation of Ltbp-4L-2xStrep and Ltbp-4S-2xStrep. Ltbp-4L-2xStrep was unaffected, whereas Ltbp-4S-2xStrep showed a shift towards lower molecular weight positions. Lower panel: Ltbp-4S-2xStrep was digested with PNGase F under native (left lanes) and denaturing (right lanes) conditions. Both conditions resulted in a shift towards lower molecular weight positions. (F,G) After digest under non-denaturing conditions Ltbp-4L-2xStrep and Ltbp-4S-2xStrep were both able to bind to rfibulin-4 and -5 immobilized on a Biacore chip. Ltbp-4L-2xStrep binding was not affected, whereas Ltbp-4S-2xStrep showed an increase in binding of 15% to 20% after deglycosylation. The continuous lines represented the response before and the dashed lines after deglycosylation (*n*=2).

The N-terminal region of Ltbp-4S directly interacts with fibulin-5 (Noda et al., 2013). Based on our data showing that at least Ltbp-4L is required for fibulin-4 deposition (Fig. 3B,E), we performed protein-protein interaction studies using N-terminal Ltbp-4L (Ltbp-4L-2xStrep) and Ltbp-4S (Ltbp-4S-2xStrep) fragments conjugated to a 2xStrep tag (Fig. 4A) and recombinant full-length fibulin-4 and fibulin-5 (rfibulin-4 and rfibulin-5). Surface plasmon resonance analysis revealed that Ltbp-4L-2xStrep and Ltbp-4S-2xStrep interact with rfibulin-4 and rfibulin-5 (Fig. 4B,C). However, the molecular dissociation of Ltbp-4L-2xStrep from both rfibulins was significantly slower than that of Ltbp-4S-2xStrep (supplementary material Table S2), indicating that the 126 N-terminal amino acids specific to Ltbp-4L are responsible for a stronger binding affinity of Ltbp-4L-2xStrep to rfibulin-4 and rfibulin-5.

### Ltbp-4 isoform-specific N-glycosylation modulates fibulin binding

*In silico* analysis revealed one putative N-glycosylation site within the specific N-terminal amino acid sequence of Ltbp-4S whereas the N-terminus of Ltbp-4L was predicted to only be subject to O-linked glycosylation (Fig. 4A). We verified N-linked glycosylation in a PNGase F deglycosylation assay with recombinant full-length human LTBP-4S (Fig. 4D; rLTBP-4S) and also with Ltbp-4S-2xStrep (Fig. 4E). PNGase F digest of Ltbp-4L-2xStrep showed no difference in band retardation (Fig. 4E). In order to test whether N-glycosylation of Ltbp-4L-2xStrep and Ltbp-4S-2xStrep contributes to rfibulin-4 and rfibulin-5 binding, the PNGase F deglycosylation assay was also performed under non-denaturating conditions to enable subsequent surface plasmon resonance analysis (Fig. 4E).

We demonstrated enhanced binding of Ltbp-4S-2xStrep to rfibulin-4 and rfibulin-5 after deglycosylation with a signal increase of 15% to 20% whereas binding between Ltbp-4L-2xStrep and rfibulin-4 or rfibulin-5 was not changed after deglycosylation (Fig. 4F,G).

## DISCUSSION

The human gene involved in ARCL1C was discovered based on the ultrastructural similarity of elastic fiber defects exhibited in mice lacking Ltbp-4S (*Ltbp4S*^−/−^) and a subcohort of cutis laxa patients (Sterner-Kock et al., 2002; Urban et al., 2009). However, unlike ARCL1C patients, *Ltbp4S*^−/−^ mice live to adulthood despite severe elastic fiber defects (Sterner-Kock et al., 2002), whereas ARCL1C patients die very early due to extensive developmental abnormalities in multiple visceral organs (Urban et al., 2009). These phenotypic differences are likely due to the inactivation of both LTBP-4 isoforms in ARCL1C patients, which does not occur in *Ltbp4S*^−/−^ mice. To test this hypothesis we generated *Ltbp4*-knockout (*Ltbp4*^−/−^) mice lacking both Ltbp-4 isoforms.

*Ltbp4*^−/−^ mice grew significantly slower than their WT littermates, developed dermal and cardiopulmonary abnormalities and died within 2 weeks after birth, most likely due to respiratory failure caused by extensive pulmonary emphysema similar to that observed in ARCL1C patients (Table 2) (Callewaert et al., 2013; Urban et al., 2009).

**Table 2.**
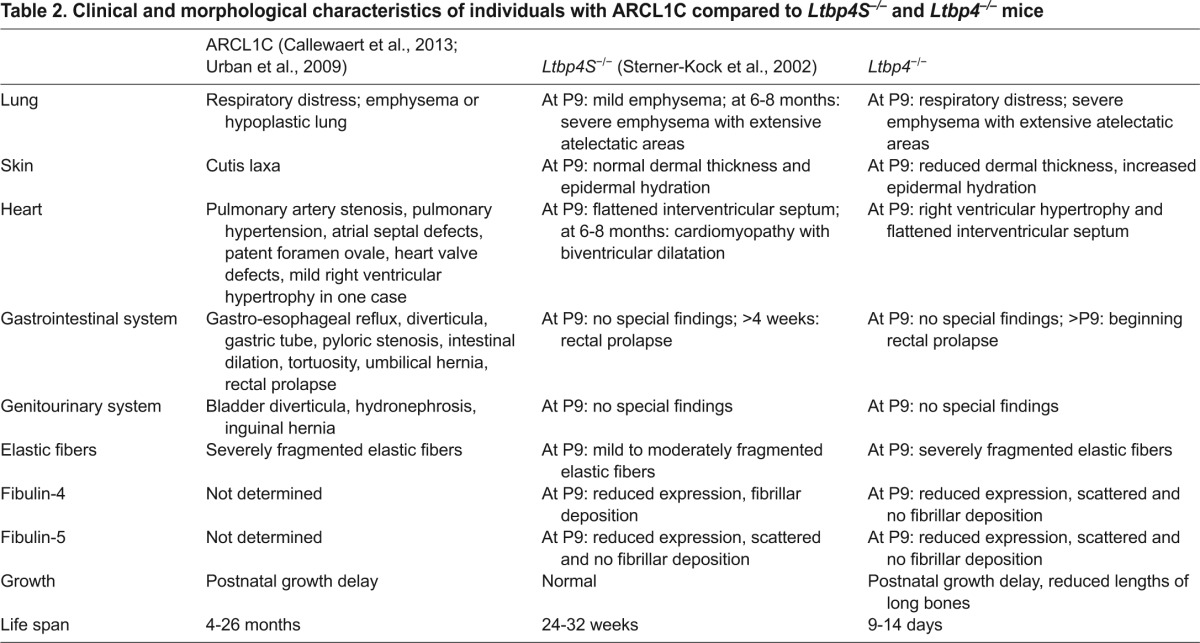
Clinical and morphological characteristics of individuals with ARCL1C compared to *Ltbp4S*^−/−^ and *Ltbp4*^−/−^ mice

The observed lung, aortic, skin and cardiac phenotype of *Ltbp4S*^−/−^ and *Ltbp4*^−/−^ mice was more severe in the *Ltbp4*^−/−^ mice (Table 2), suggesting that Ltbp-4L compensates for some Ltbp-4S functions. Ltbp-4L functions seem particularly important during postnatal development and survival to adulthood (Sterner-Kock et al., 2002). For instance, in the skin, the relatively low Ltbp-4L expression compared with Ltbp-4S seems to be important for dermal thickness and density. The distinct tissue expression patterns of Ltbp-4L in heart and aorta might result from the activation of the two known Ltbp-4 promoters under the control of independent transcription factors (Bultmann et al., 2013; Kantola et al., 2010).

*Ltbp4*^−/−^ mice displayed right ventricular hypertrophy in the heart, which *Ltbp4S*^−/−^ mice do not. This could be either a primary consequence caused by the additional loss of Ltbp-4L or secondary to increased pulmonary resistance leading to pulmonary hypertension (Bleeker et al., 2006). The human ARCL1C syndrome includes pulmonary artery stenosis, pulmonary hypertension, atrial septal defects, patent foramen ovale, heart valve defects and a mild right ventricular hypertrophy in one case (Urban et al., 2009). In contrast to older *Ltbp4S*^−/−^ mice (Sterner-Kock et al., 2002), no cardiomyopathy was found in ARCL1C patients, but these conditions might require a longer time to develop in humans (Urban et al., 2009).

The disruption of the aortic IEL in mice seems to be dependent on the expression of Ltbp-4L because we found that only *Ltbp4*^−/−^ but not *Ltbp4S*^−/−^ mice had severely disrupted IEL. Fibulin-2 and fibulin-5 have been reported to function cooperatively to form the IEL during postnatal development (Chapman et al., 2010). Interestingly, Ltbp-4 deficiency leads to upregulation of fibulin-2, especially in the area of the endothelial lining of the vessel walls in the heart (supplementary material Fig. S7). Obviously, this upregulation of fibulin-2 could protect the IEL only in *Ltbp4S*^−/−^ mice, indicating a possible direct or indirect interaction between Ltbp-4L and fibulin-2. Similar to other models of disturbed elastogenesis, including elastin (*Eln*^−/−^) (Li et al., 1998), fibulin-4 (*Fibulin-4*^−/−^ and *Fibulin-4*^R/R^) (Hanada et al., 2007; McLaughlin et al., 2006), and lysyl oxidase (*Lox*^−/−^) (Mäki et al., 2005) mutant mice, *Ltbp4*^−/−^ and to a lesser extent *Ltbp4S*^−/−^ mice develop thick aortic walls. Ultrastructural analysis of aortas (data not shown) and elastic lung arteries (supplementary material Fig. S5) revealed the presence of amorphous material between elastic fibers in *Ltbp4*^−/−^ mice, a phenotype that has already been described in *Fibulin-4*^R/R^ mice (Hanada et al., 2007). However, we were not able to characterize this material in more detail.

Fragmented elastic fibers are the hallmark of LTBP-4 deficiency in both humans and mice (Sterner-Kock et al., 2002; Urban et al., 2009). *Ltbp4*^−/−^ mice and ARCL1C patients (Callewaert et al., 2013; Urban et al., 2009) show massive elastic fiber fragmentation, which is significantly less severe in *Ltbp4S*^−/−^ mice. Formation of functional elastic fibers requires cross-linking of tropoelastin monomers after oxidative deamination by lysyl oxidases and organization of the insoluble elastin matrix onto a pre-assembled fibrillin microfibril scaffold (Mithieux and Weiss, 2005; Wagenseil and Mecham, 2007; Yanagisawa and Davis, 2010). In addition to structural proteins and cross-linking enzymes, elastic fiber formation requires multiadhesive adaptor proteins, such as fibulin-4 (Horiguchi et al., 2009; McLaughlin et al., 2006), fibulin-5 (Nakamura et al., 2002; Yanagisawa et al., 2002) and Ltbp-4 (Noda et al., 2013; Sterner-Kock et al., 2002; Urban et al., 2009), all of which are present on microfibrils. Fibulin-4 and fibulin-5 have crucial non-redundant functions in the assembly of elastic fibers (Papke and Yanagisawa, 2014).

Fibulin-5 mediates coacervation of tropoelastin prior to cross-linking (Hirai et al., 2007; Wachi et al., 2008) by binding to Ltbp-4S (Noda et al., 2013), whereas fibulin-4 enhances the binding of tropoelastin to lysyl oxidase and thus facilitates cross-linking (Choudhury et al., 2009; Horiguchi et al., 2009). *Fibulin-5*^−/−^ mice have severely disorganized elastic fibers throughout the body and develop severe and progressive elastinopathy postnatally, but survive to adulthood (Nakamura et al., 2002; Yanagisawa et al., 2002). *Fibulin-4*^−/−^ mice fail to form elastic fibers and die perinatally (Horiguchi et al., 2009; McLaughlin et al., 2006), whereas hypomorphic *Fibulin-4*^R/R^ mice can survive up to 14 weeks (Hanada et al., 2007). This suggests that at least 25% fibulin-4 expression, and probably its proper deposition, but not fibulin-5, is crucial for postnatal survival. The expression of fibulin-5 and to a lesser extent fibulin-4 was repressed in *Ltbp4S*^−/−^ and *Ltbp4*^−/−^ mice, suggesting that Ltbp-4L and Ltbp-4S are required for adequate fibulin-4 and fibulin-5 expression. The molecular mechanisms leading to fibulin-4 and fibulin-5 repression in Ltbp-4-deficient mice remain to be investigated. This repression could possibly be mediated by an outside-in signaling cascade triggered by cellular sensing of an altered ECM in Ltbp-4-deficient mice.

The fibrillar deposition of fibulin-5 is destroyed in both Ltbp-4-deficient mouse strains. However, matrix deposition of fibulin-4 appeared to be unaltered in *Ltbp4S*^−/−^ mice, whereas it was highly defective in *Ltbp4*^−/−^ mice, suggesting that Ltbp-4L is important for this process. The ECM deposition of other ECM proteins, such as fibronectin and fibrillin-1, was not affected by Ltbp-4 deficiency (supplementary material Fig. S8). We showed that Ltbp-4L and Ltbp-4S bind to fibulin-4 and fibulin-5 through their N-termini with different affinities. The much higher affinity of Ltbp-4L to fibulin-4 and fibulin-5 might reflect its importance during early postnatal development when rapid elastic fiber formation is mandatory, for example, during postnatal lung maturation. A possible mechanism to modify protein-protein binding is the attachment of glycosaminoglycan (GAG) chains (Lowery et al., 2014; Torreno-Pina et al., 2014). We verified the predicted specific N-glycosylation of Ltbp-4S *in vitro* and *in vivo* (supplementary material Fig. S9) and showed that deglycosylation enhances binding of Ltbp-4S but not of Ltbp-4L to fibulin-4 and fibulin-5. N-glycosylation patterns can vary between different tissues and thereby possibly modulate Ltbp-4S binding to fibulin-4 and fibulin-5. In situations of high glycosylation of the Ltbp-4S N-terminus, Ltbp-4L-driven binding mechanisms might be favored. However, another possibility is that this specific N-glycosylation site is used to enable Ltbp-4S to bind to additional unknown factors.

Besides their obvious role within the ECM, Ltbp-4L and Ltbp-4S are reported to modulate the activity of TGFβ by facilitating its secretion and deposition into the ECM (Annes et al., 2004; Miyazono and Heldin, 1991). However, TGFβ activity was not affected in lung fibroblasts of the two *Ltbp4*-knockout strains (supplementary material Fig. S10), suggesting that the observed phenotypes are largely attributable to the loss of Ltbp-4 structural functions.

**Fig. 5.**
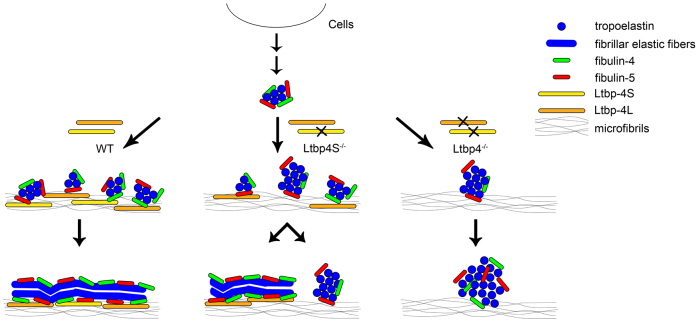
**Proposed model for the role of Ltbp-4L and Ltbp-4S in elastogenesis.** In the presence of Ltbp-4L and Ltbp-4S, microaggregation of tropoelastin, which is tethered to fibulin-4 and fibulin-5, deposits linearly onto microfibrils. Subsequent coalescence of tropoelastin takes place on microfibrils, resulting in fibrillar deposition of the tropoelastin–fibulin-4–fibulin-5 complex. In the absence of Ltbp-4S, the tropoelastin–fibulin-4–fibulin-5 complex only partly deposits on microfibrils, resulting in scattered fibrillar elastic fibers as well as amorphous aggregates of tropoelastin–fibulin-4–fibulin-5, which grow to form globular structures. In the absence of Ltbp-4L and Ltbp-4S, all tropoelastin–fibulin-4–fibulin-5 complexes form dysmorphous, non-fibrillar globular structures. Modified from Noda et al. (Noda et al., 2013).

Our results suggest that Ltbp-4L and Ltbp-4S orchestrate elastin deposition onto the ECM fibrillin microfibrils by binding fibulin-4 and fibulin-5, which in turn both target tropoleastin (Choudhury et al., 2009; Nakamura et al., 2002). In the absence of Ltbp-4S, the tropoelastin–fibulin-4–fibulin-5 complexes are deposited only partially onto microfibrils, resulting in an ECM with scattered fibrillar elastic fibers surrounded by amorphous tropoelastin-fibulin aggregates. In ECMs produced by cells lacking Ltbp-4L and Ltbp-4S, fibrillar fibers are non-existent and only amorphous aggregates are observed, strongly suggesting that both Ltbp-4 isoforms are required for normal elastogenesis (Fig. 5).

In summary, we characterized a new mouse model for ARCL1C that will help to elucidate the pathogenesis of this severe disease. We further showed that Ltbp-4L and Ltbp-4S have important and partially non-overlapping roles in survival and elastic fiber formation, and identified fibulin-4 as a new interacting partner of both Ltbp-4 isoforms. Moreover, we showed that specific N-glycosylation of Ltbp-4S influences its fibulin-4- and fibulin-5-binding affinity. Future experiments involving the generation of *Ltbp4L*- and tissue-specific-*Ltbp4*-knockout mouse lines are planned to gain further insights into the isoform-specific functions of Ltbp-4.

## MATERIALS AND METHODS

### Animals, breeding and genotyping

The embryonic stem cell clone E301B04 (E14TG2a.4, 129P2) carrying a gene-trap insertion in the fifth intron of the *Ltbp4* gene (supplementary material Fig. S1) was purchased from the German Gene Trap Consortium (GGTC, Helmholtz-Zentrum München, Munich, Germany). These cells were used to derive chimeric mice that were mated with C57BL/6N female mice. The offspring was tested for transgene germline transmission and backcrossed to C57BL/6N background for ten generations. Genotyping of *Ltbp4*-knockout mice was performed using 5mL4_gen, 3mL4_gen and betageo_gen primers (supplementary material Table S3) complementary to the sequences flanking the gene-trap insertion sites. Generation and genotyping of *Ltbp4S*^−/−^ mice was performed as described previously (Sterner-Kock et al., 2002). Animals were housed in a 12-hour-light-12-hour-dark cycle and were fed a standard rodent diet (ssniff Spezialdiäten, Soest, Germany).

Unless otherwise stated, all animals were sacrificed at postnatal day 9 (P9) by decapitation and autopsies were carried out using standard protocols. For blood gas analyses (BGA), trunk blood was collected in a heparinised 1.5 ml tube and pH, pCO_2_, pO_2_, sO_2_, *ct*HB, Hct, HCO_3_ and ABE were analyzed using an ABL800 Flex blood gas analyzer (Radiometer, Willich, Germany). All animal procedures were performed in accordance with the German Laws for Animal Protection and were approved by the Institutional Animal Care and Use Committee: Landesamt für Natur, Umwelt und Verbraucherschutz Nordrhein-Westfalen (Recklinghausen, Germany).

### mRNA expression analysis

Total RNA isolation and real-time PCR were performed as described previously (Bultmann et al., 2013). All primers and probes are listed in supplementary material Table S3. Quantification of mRNA levels for *Ltbp4*, *Ltbp4S* and *Ltbp4L* were calculated using the standard curve method (Bultmann et al., 2013). Relative expression of tropoelastin, fibulin-5 and fibulin-4 was adjusted for total RNA content by normalizing to glyceraldehyde-3-phosphate dehydrogenase (Gapdh) expression. Calculations were performed by a comparative 2^−ΔΔCT^ method (Livak and Schmittgen, 2001).

### SDS-PAGE and immunoblotting

Protein expression levels were determined by western blotting, using SDS-PAGE as described previously (Bultmann et al., 2013). All antibodies used are listed in supplementary material Table S4.

### Histology and immunohistochemistry

Mice were sedated with ketamine and xylazine. The heart was punctured and perfused with PBS and 4% paraformaldehyde according to standard protocols. Perfusion-fixed aortas, lungs and hearts were dissected and embedded in paraffin. 4-μm sections were stained with haematoxylin-eosin, resorcin-fuchsin (elastin) or alcian blue (glucosaminoglycans). For immunohistochemistry, tissue sections were deparaffinized and antigen retrieval was performed in 10 mM citrate puffer (pH 6.0) for 20 minutes in a microwave (Ltbp4, PCNA) or by using proteaseXXIV (DCS Innovative Diagnostik-Systeme, Hamburg, Germany) for 6 minutes at room temperature (fibulin-4, fibulin-5) followed by treatment with 3% H_2_O_2_ for 15 minutes at room temperature. Incubation with primary antibodies (supplementary material Table S4) in dilution buffer was performed for 1 hour at room temperature. For detection, Supervision 2-Single Species HRP Polymer (DCS Innovative Diagnostik-Systeme, Hamburg, Germany) and Permanet AEC-Kit (ZYTOMED Systems, Berlin, Germany) were used according to manufacturers’ protocols.

### Analyses of skin parameters

The epidermal and dermal thicknesses were determined in skin sections. Epidermal hydration, transepidermal water loss and net-elasticity of the skin were analyzed using the Multi Probe Adapter (MPA) System (Courage+Khazaka electronic, Cologne, Germany) with the Corneometer CM 825-, Tewameter TM 300- or Cutometer MPA 580-probe, respectively, and freshly sacrificed mice according to the manufacturer’s instructions.

### Stereological analyses of the cardiac ventricles

The stereological analyses of the cardiac ventricles were determined in myocardial sections fixed *in situ* and stained with haematoxylin-eosin as described previously (Hilfiker-Kleiner et al., 2004; Ricke-Hoch et al., 2014).

### Echocardiography of neonatal mice

High-resolution mouse echocardiography was performed in WT and *Ltbp4*^−/−^ mice using a commercially available ultrasound system (Philips iE33, Philips, Hamburg, Germany) equipped with a 15 MHz linear array transducer utilizing harmonic imaging (L15-io7, Philips, Hamburg, Germany). Volumetric analysis of left-ventricular structures was performed to assess left-ventricular morphology and function (Ghanem et al., 2006; Zheng-Fischhöfer et al., 2006).

### Magnetic resonance imaging of neonatal mice

Magnetic resonance imaging (MRI) studies were performed in two mice per genotype using a clinical 3 Tesla MR whole-body system (Achieva 2.3, Philips, Best, The Netherlands) as described previously (Bunck et al., 2009). For optimal MR signal detection, a dedicated solenoid receive-only coil was used (Philips, Forschungslaboratorien, Aachen, Germany). After positioning of the mouse in the isocenter of the magnet gradient, echo scout images were acquired in a horizontal orientation for localization of the heart. For cine imaging, we used a retrospectively ECG-triggered, segmented gradient echo sequence. The number of signal averages was set to eight to achieve good signal-to-noise ratios. The reconstructed in-plane spatial resolution was 0.13×0.13 mm; slice thickness was set to 0.8 mm. Per heart cycle, 30 phases were imaged. For left-ventricular assessment, standard imaging planes were obtained according to the recommendations by Schneider et al. (Schneider et al., 2006).

### Electron microscopy

For transmission electron microscopy, organ tissues were fixed in 5% glutaraldehyde in 0.4 M PBS buffer (pH 7.2–7.3). The samples were washed three times with 0.1 M cacodylate buffer and stored in this buffer at 4°C until plastic embedding. Afterwards preparations were postfixed with 2% osmium tetraoxide in 0.1 M cacodylate buffer for 2 hours at 4°C. Before embedding in araldite (Novartis Pharma, Nürnberg, Germany) the specimens were dehydrated in a graded series of ethanol. Sections of plastic-embedded specimens were cut with a diamond knife for thin sections and ultrathin sections on an ultra-microtome (Reichert, Buffalo, NY, USA). The 0.5-μm thin slices were stained with Methylene Blue and examined using a Zeiss Axiovert 200 (Zeiss, Oberkochen, Germany). Ultrathin sections (60 nm) were mounted on formvar-coated copper grids, stained with 0.2% uranyl acetate and lead citrate and then examined with a Zeiss EM 902 A electron microscope (Zeiss, Oberkochen, Germany).

### Isolation of elastin, and quantification of desmosine and isodesmosine

Pure elastin, free of contaminants by other ECM components, was isolated from biopsies of mouse lung as described previously (Schmelzer et al., 2012). Isolated elastin samples were weighed and then hydrolyzed at a concentration of 1 mg/ml in 6 M HCl at 105°C for 24 hours, respectively, to allow the liberation of DES and IDES. After incubation, the samples were evaporated to dryness at 60°C, and the residues were taken up in acetonitrile:water (1:1, v/v) prior to liquid chromatography mass spectrometry (LC-MS) analysis. LC-MS analysis of DES and IDES was carried out using an Agilent 1100 LC system (Agilent Technologies, Waldbronn, Germany) coupled to a quadrupole ion trap mass spectrometer Finnigan LCQ (Thermo Fisher Scientific, Waltham, MA) by an electrospray interface. Isocratic chromatographic separation was performed over 8 minutes at a flow rate of 0.2 ml/min on a Reprosil-Pur 120 C18 AQ 3 μm column (150×2 mm, Maisch, Ammerbuch-Entringen, Germany). 0.1% formic acid/methanol (97.5:2.5, v/v) was used as the mobile phase and the column temperature was 40°C. Under these chromatographic conditions DES and IDES co-elute. For mass spectrometric analyses, the following parameters were used: positive ion mode, electrospray voltage: 4.5 kV, heated capillary temperature: 220°C.

### Isolation of primary lung fibroblasts

Mouse lung fibroblasts were isolated according to described protocols (Seluanov et al., 2010). In brief, tissues were finely chopped, and digested with 0.12 Wünsch units/ml Liberase TM research Grade (Roche Diagnostics, Mannheim, Germany) for 30 minutes at 37°C. Cells were outgrown in DMEM/F12 (Gibco, Life Technologies, Darmstadt, Germany) containing 15% FCS (Biochrom, Berlin, Germany) and antibiotic/antimycotic (Life Technologies, Darmstadt, Germany) at 37°C in 5% CO_2_. After the first passage, remaining tissue fragments were discarded and cells were incubated in EMEM (Gibco, Life Technologies, Darmstadt, Germany) containing 15% FCS and penicillin-streptomycin (Life Technologies, Darmstadt, Germany).

### Expression of recombinant proteins and deglycosylation assay

Full-length rLTBP-4S was purified as previously described (Noda et al., 2013). The first 253 amino acids of mouse Ltbp-4L (Ser25-Asp277) and the first 185 amino acids of mouse Ltbp-4S (Arg27-Asp211) after the signal peptide, as well as mouse full-length fibulin-4 and fibulin-5 after the signal peptide, were cloned into a modified pCEP-Pu vector containing a 3ʹ 2xStrepII tag using the restriction sites *Nhe*I and *Xho*I, and stably expressed in human embryonic kidney (HEK) 293 EBNA cells. The Strep-tagged proteins were purified from conditioned medium. Collected supernatants were supplemented with 1 mM phenylmethylsulfonyl fluoride, filtered and passed over a streptactin-Sepharose column. The recombinant proteins were eluted with elution buffer (100 mM Tris-HCl pH 7.4, 150 mM NaCl) containing 2.5 mM d-desthiobiotin.

Deglycosylation of denatured rLTBP-4S (200 ng) or homogenized WT lung (20 μg) was performed using 250 units recombinant PNGase F (New England Biolabs, Frankfurt am Main, Germany) for 3 hours at 37°C, or of native or denatured Ltbp-4L-2xStrep and Ltbp-4S-2xStrep (10 μg) using 500 units of PNGase F for 48 hours at 37°C according to the manufacturer’s protocol.

### Immunofluorescence analysis

Primary lung fibroblasts were seeded on glass coverslips and cultured for 7 days in EMEM, 15% FCS and penicillin-streptomycin with or without recombinant full-length fibulin-4 (20 nm). Cells were fixed in methanol or PFA for 20 minutes at −20°C, and blocked with 5% BSA in PBS for 1 hour at room temperature. Cells were incubated with primary antibodies (supplementary material Table S4) in 1% BSA overnight at 4°C. Secondary antibodies were coupled to Alexa-Fluor-488 donkey anti-rabbit IgG, or Alexa-Fluor-546 donkey anti-goat IgG (Life Technologies, Darmstadt, Germany). Nuclei were counterstained with DAPI (Life Technologies, Darmstadt, Germany). Images were taken using a confocal LSM710 microscope (Zeiss, Oberkochen, Germany).

### Surface plasmon resonance

Binding analyses were performed by using a BIAcore2000 (GE Healthcare GmbH, Solingen, Germany). 570 RUs of rfibulin-4, and 390 RUs of rfibulin-5 were covalently coupled to carboxymethyldextran hydrogel 500M sensor chips (XanTec, Düsseldorf, Germany) using the amine coupling kit following the manufacturer’s instructions (GE Healthcare, Solingen, Germany). Interactions between Ltbp-4L-2xStrep or Ltbp-4S-2xStrep and rfibulin-4 and rfibulin-5 were performed as previously described (Sengle et al., 2008). For determination of the kinetic constants, each individual sensorgram was fitted using the BIAevaluation 4.1 software (1:1 Langmuir interaction model) according to the manufacturer’s instructions. The determined pseudo-first order kinetic constants *k*_obs_ were plotted versus the analyte concentrations and a linear best fit was applied. The average *k*_off_ equals the intersection with the *y*-axis. The slope of the fitted straight line is a measure of the *k*_on_ rate. Apparent equilibrium dissociation constants (*K*_d_) were then calculated as the ratio of *k*_off_ to *k*_on_.

### TGFβ activity bioassay

TGFβ levels were assessed in 48-hour conditioned medium of primary lung fibroblasts that were cultured in DMEM (2% FCS, 1% penicillin-streptomycin, 10% BSA). TGFβ activity was determined by using a plasminogen activator inhibitor-1 (PAI-1) promoter luciferase reporter assay (Abe et al., 1994) using mink lung epithelial cells stably expressing the firefly luciferase reporter gene under the control of a TGFβ-specific, truncated PAI-1 promoter_._

Active TGFβ was determined after overnight incubation of the reporter cells with conditioned medium or with recombinant human TGFβ1 standards. The reporter cells were lysed and luciferase activity was measured using the Beetle-Juice kit (PJK, Kleinblittersdorf, Germany) and a GloMax-Multi Detection System (Promega, Mannheim, Germany).

### Statistical evaluation

Data are presented as a mean±s.d. Differences between groups were analyzed by Mann-Whitney test, log-rank test, Student’s *t*-test or ANOVA, followed by Bonferroni correction as appropriate. Statistical significance of post-hoc analyses was taken at *P*<0.05. Calculations were performed using SPSS21 (IBM Deutschland, Ehningen, Germany).

## Supplementary Material

Supplementary Material
