# Supplementary material for: Modeling autosomal recessive cutis laxa type 1C in mice reveals distinct functions for Ltbp-4 isoforms
Source: Dis Model Mech. 2015 Feb 20;8(4):403–15. doi: 10.1242/dmm.018960 (PMC4381339; doi:10.1242/dmm.018960)
Supplement: Supplementary Material [file supp_8_4_403__index.html]

Modeling autosomal recessive cutis laxa type 1C in mice reveals distinct functions for Ltbp-4 isoforms — Supplementary Material 

# Modeling autosomal recessive cutis laxa type 1C in mice reveals distinct functions for Ltbp-4 isoforms

## DMM018960 Supplementary Material

**Files in this Data Supplement:**

- **Supplementary Material**
