# Supplementary material for: Modeling autosomal recessive cutis laxa type 1C in mice reveals distinct functions for Ltbp-4 isoforms
Source: Dis Model Mech. 2015 Feb 20;8(4):403–15. doi: 10.1242/dmm.018960 (PMC4381339; doi:10.1242/dmm.018960)
Supplement: Supplementary Material [file supp_8.4.403_DMM018960.pdf]

Modeling Autosomal Recessive Cutis Laxa Type 1C (ARCL1C) in Mice Reveals Distinct Functions of Ltbp-4 Isoforms

Running title: Ltbp-4L and Ltbp-4S in ARCL1C

Insa Bultmann-Mellin[1¶], Anne Conradi[1¶], Alexandra C. Maul[1], Katharina Dinger[1,2], Frank Wempe[3], Alexander P. Wohl[4], Thomas Imhof[4,5], F. Thomas Wunderlich[6,7,8], Alexander C. Bunck[9], Tomoyuki Nakamura[10], Katri Koli[11], Wilhelm Bloch[12], Alexander Ghanem[13], Andrea Heinz[14], Harald von Melchner[3], Gerhard Sengle[4,6], and Anja Sterner-Kock[1]*

[1]Center for Experimental Medicine, Medical Faculty, University of Cologne, Cologne, Germany
[2]Department of Pediatrics and Adolescent Medicine, Medical Faculty, University of Cologne, Cologne, Germany
[3]Department of Molecular Hematology, University of Frankfurt Medical School, Frankfurt am Main, Germany
[4]Center for Biochemistry, Medical Faculty, University of Cologne, Cologne, Germany
[5]Institute for Dental Research and Oral Musculoskeletal Biology, Medical Faculty, University of Cologne, Cologne, Germany
[6]Center for Molecular Medicine Cologne (CMMC), University of Cologne, Cologne, Germany
[7]Max Planck Institute for Metabolism Research, Cologne, Germany
[8]Cologne Excellence Cluster on Cellular Stress Responses in Aging-Associated Diseases (CECAD), University of Cologne, Cologne, Germany
[9]Department of Radiology, Medical Faculty, University of Cologne, Cologne, Germany
[10]Department of Pharmacology, Kansai Medical University, Osaka, Japan
[11]Research Programs Unit and Transplantation Laboratory, Haartman Institute, University of Helsinki, Helsinki, Finland
[12]Institute of Cardiology and Sports Medicine, German Sport University Cologne, Cologne, Germany
[13]Department of Medicine/Cardiology, University of Bonn, Bonn, Germany
[14]Institute of Pharmacy, Martin Luther University Halle-Wittenberg, Halle (Saale), Germany

*Corresponding author

E-mail: anja.sterner-kock@uk-koeln.de (ASK)

[¶]These authors contributed equally to this work

Keywords: Latent Transforming Growth Factor Beta Binding Protein 4, Ltbp-4, Ltbp-4L, Ltbp-4S, Autosomal Recessive Cutis Laxa Type 1C, ARCL1C, Elastogenesis, Extracellular Matrix, ECM, Fibulin-4, Fibulin-5

Supplementary material

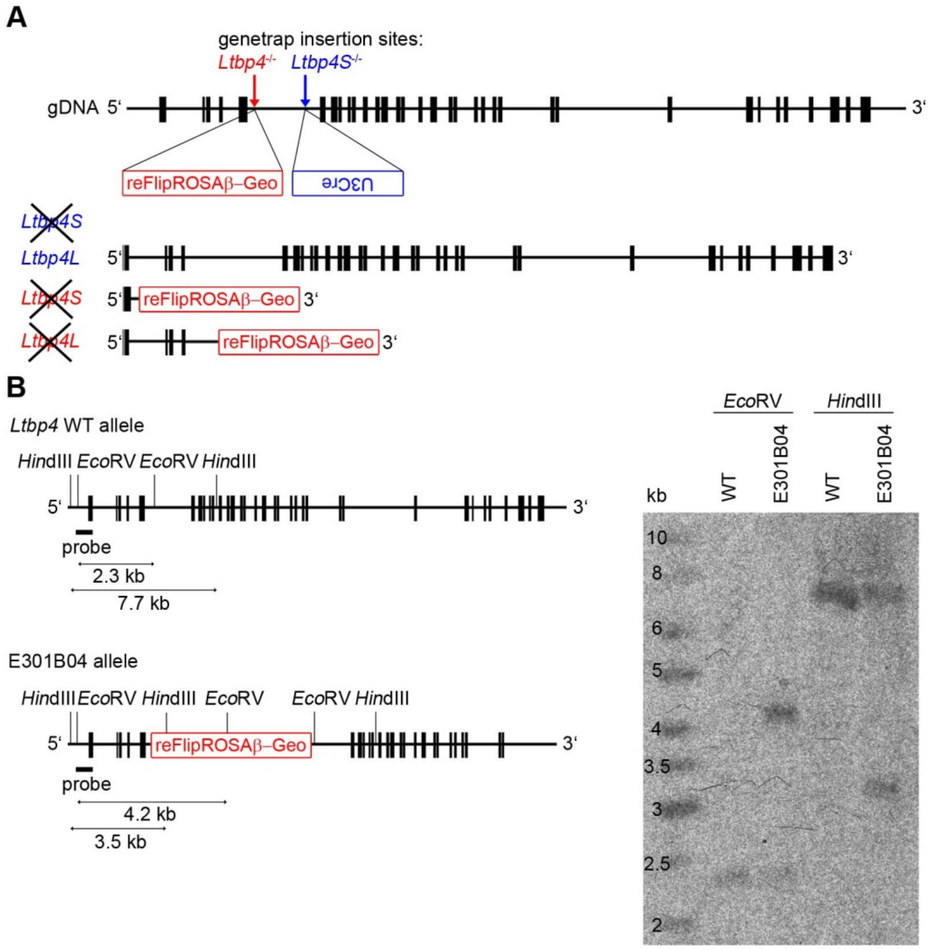

Supplementary material Fig. S1. Generation of *Ltbp4S*$^{-/-}$ and *Ltbp4*$^{-/-}$ mice.

(A) To generate mice lacking Ltbp-4 we obtained an ES-cell clone (E301B04) from GGTC (German Gene Trap Consortium, Germany), containing a FlipROSAβgeo gene-trap cassette in the first intron of the short isoform of *Ltbp4* (*Ltbp4S*; e.g. in the fourth intron of *Ltbp4L*). The used vector consisted of a promoterless βgeo-reporter with an upstream splice acceptor site (SA) and a downstream polyadenylation sequence (pA) that terminated expression of both *Ltbp4* transcripts. The integration of the U3Cre vector was described previously (Sterner-Kock et al., 2002) (gDNA= genomic DNA). (B) Representative Southern Blot analysis of gDNA from wild-type (WT) and ES-cell clone E301B04 showing mutant and WT bands using *Eco*RV- or *Hin*dIII-digested gDNA and specific 5’-probes.

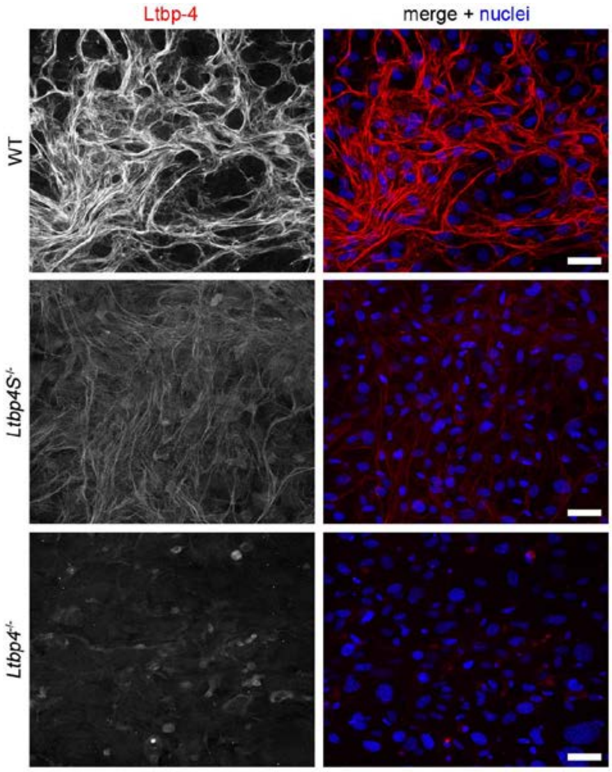

Supplementary material Fig. S2. Expression of Ltbp-4 is reduced in *Ltbp4S*$^{-/-}$ and absent in *Ltbp4*$^{-/-}$ primary lung fibroblasts.

Representative immunofluorescence staining of Ltbp-4 revealed reduced expression in primary lung fibroblasts from *Ltbp4S*$^{-/-}$ and no expression in primary lung fibroblasts from *Ltbp4*$^{-/-}$ mice compared to WT mice (Scale bar= 50 μm).

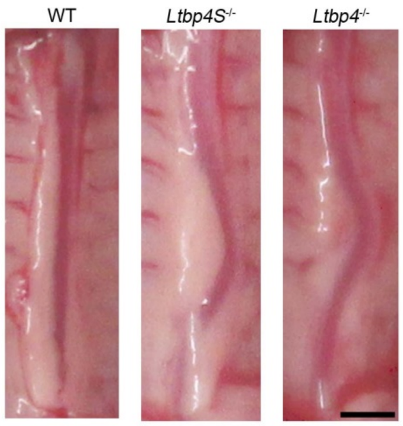

Supplementary material Fig. S3. Ltbp-4 deficient mice have tortuous aortae.

Ltbp4S$^{-/-}$ and *Ltbp4*$^{-/-}$ mice showed tortuous aortae compared to WT mice (Scale bar= 10 mm).

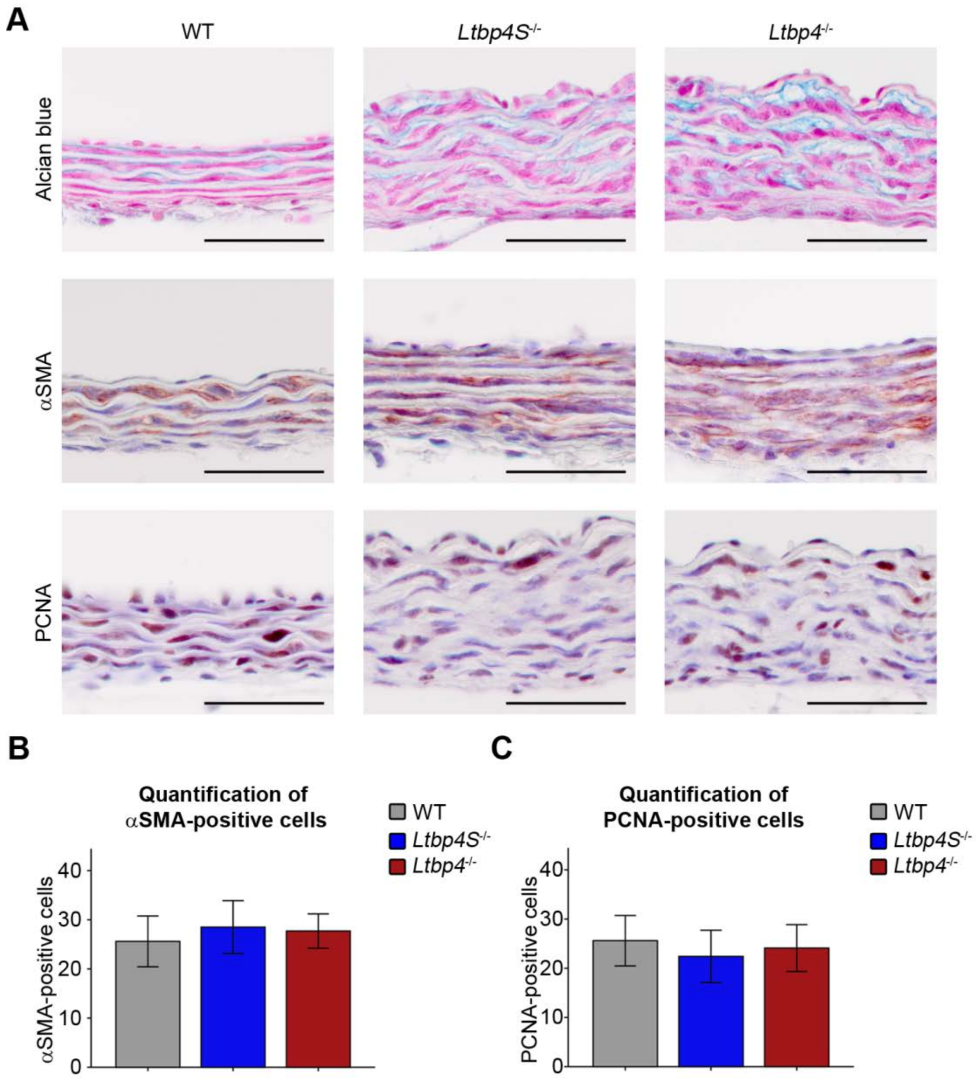

Supplementary material Fig. S4. Analysis of aortic walls in Ltbp-4 deficient mice.

(A) Aortic walls of *Ltbp4S*$^{-/-}$ and *Ltbp4*$^{-/-}$ mice showed comparable signals for glycosaminoglycans (Alcian blue), αSMA (marker for smooth muscle cells), or PCNA (marker for cellular proliferation). (B,C) There was no difference between WT, *Ltbp4S*$^{-/-}$ and *Ltbp4*$^{-/-}$ aortic walls regarding the number of (B) αSMA-positive, or (C) PCNA-positive cells. (Scale bar= 50 µm; n= 10; not significant).

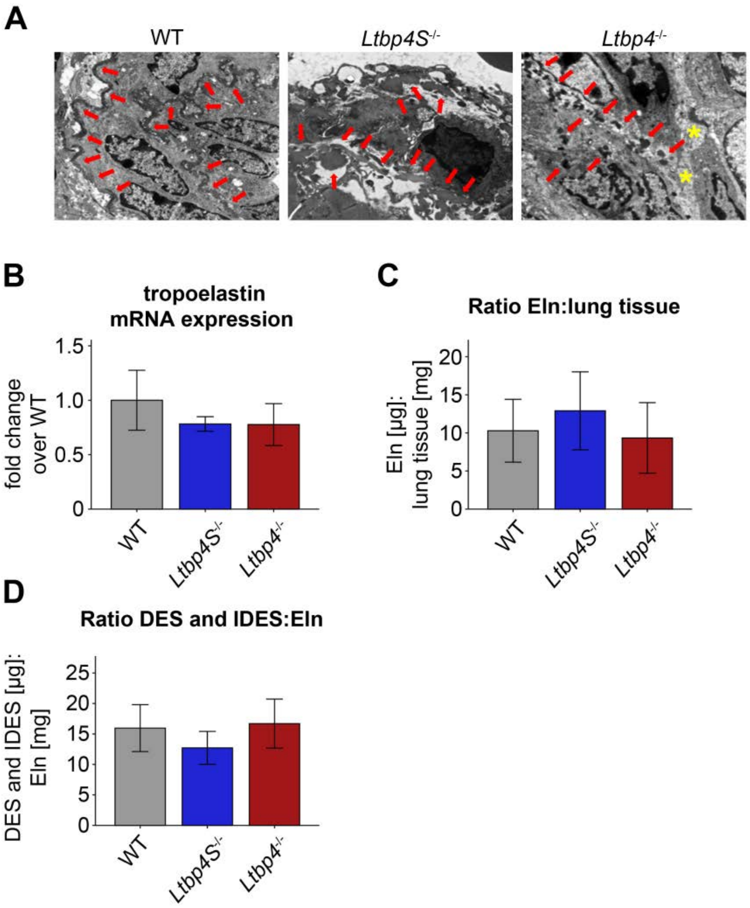

Supplementary material Fig. S5. Electron microscopy in Ltbp-4 deficient mice reveals that integrity of elastic lamellae correlates with Ltbp-4 presence, while the elastin content and cross-linking is not affected.

(A) Ultrastructural images of lungs from *Ltbp4*$^{-/-}$ mice revealed patches of condensed, amorphous elastin, scattered irregularly, lacking organized, intact elastic fibers. In *Ltbp4S*$^{-/-}$ mice short fragments of plump, amorphous elastic fiber fragments and well organized elastic fibers were evident. Red arrows point to deposited elastic material and yellow asterisks (*) to amorphous material between elastic fibers. (B) mRNA expression of tropoelastin showed no differences in lungs from *Ltbp4S*$^{-/-}$ and *Ltbp4*$^{-/-}$ mice compared to WT mice. Tropoelastin mRNA expression of WT mice was set to 1 ($n \geq 3$; not significant). (C) Comparable amounts of elastin (Eln) were presented in lung tissue from *Ltbp4S*$^{-/-}$ and *Ltbp4*$^{-/-}$ mice compared to WT mice ($n \geq 5$; not significant). (D) Ratios of desmosine and isodesmosine (DES and IDES) per mg Eln were similar in lungs from *Ltbp4S*$^{-/-}$ and *Ltbp4*$^{-/-}$ mice compared to WT mice ($n \geq 5$; not significant).

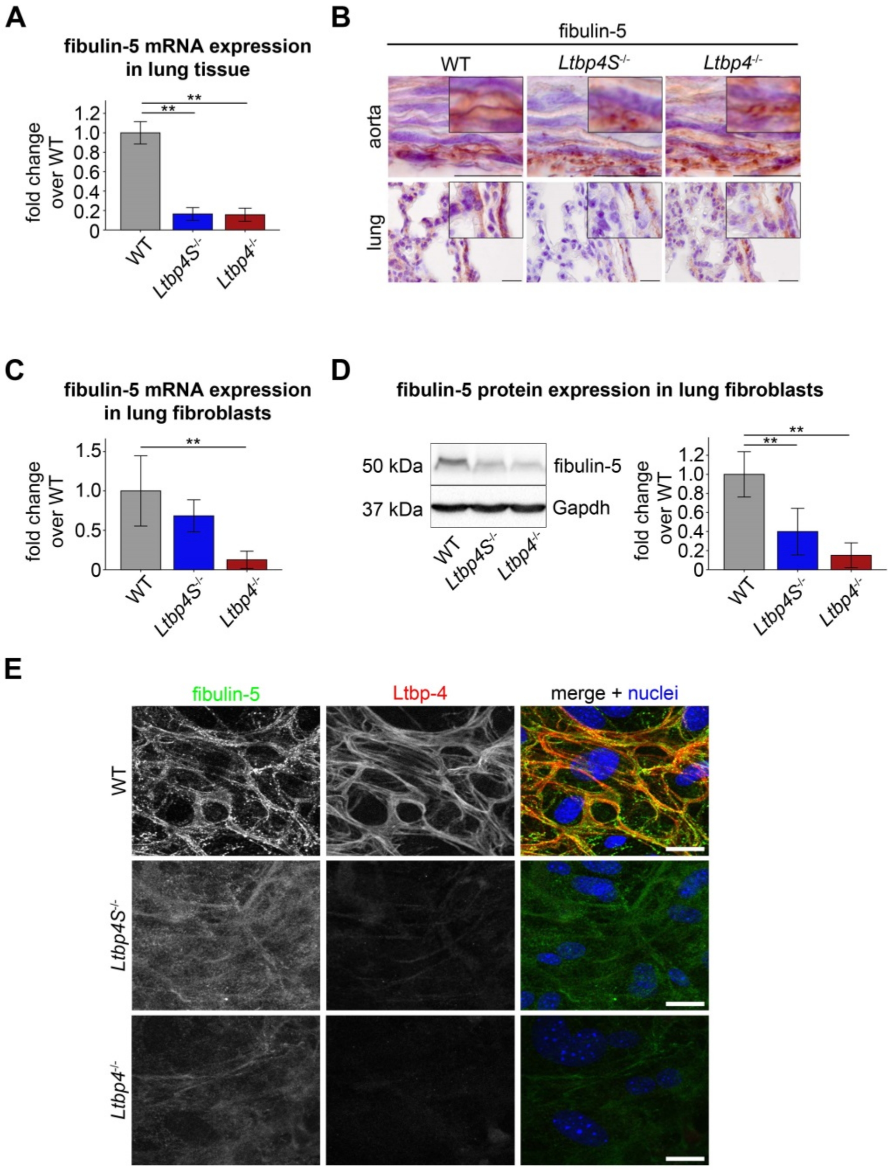

Supplementary material Fig. S6. Ltbp-4S is necessary for ECM deposition of fibulin-5.

(A) Fibulin-5 mRNA expression was significantly downregulated in lungs from *Ltbp4S*$^{-/-}$ and *Ltbp4*$^{-/-}$ mice compared to WT mice (n= 4; **p<0.01). (B) Representative images showed disruption of the fibrillar structure of fibulin-5 fibers in aortic walls (upper panel; scale bars= 20 µm) and lungs (lower panel; scale bars= 50 µm) from *Ltbp4S*$^{-/-}$ and *Ltbp4*$^{-/-}$ mice compared to WT mice. (C) Fibulin-5 mRNA expression displayed significant downregulation in lung fibroblasts isolated from *Ltbp4*$^{-/-}$ mice compared to *Ltbp4S*$^{-/-}$ and WT mice (n≥ 5; **p<0.01). (D) Representative immunoblot of lung fibroblasts (left) and its densitometric analysis (right) revealed significant downregulation of fibulin-5 in *Ltbp4S*$^{-/-}$ and *Ltbp4*$^{-/-}$ mice compared to WT mice (n≥ 4;**p<0.01). (E) Representative immunofluorescence staining of Ltbp-4 and fibulin-5 of primary lung fibroblasts revealed reduced Ltbp-4 immunoreactivity in *Ltbp4S*$^{-/-}$ and absence of Ltbp-4 in *Ltbp4*$^{-/-}$ mice. The ECM deposition of fibulin-5 was impaired in *Ltbp4S*$^{-/-}$ and *Ltbp4*$^{-/-}$ mice compared to WT mice. Scale bars= 100 µm. Protein as well as mRNA expression of fibulin-5 of the WT was set to 1.

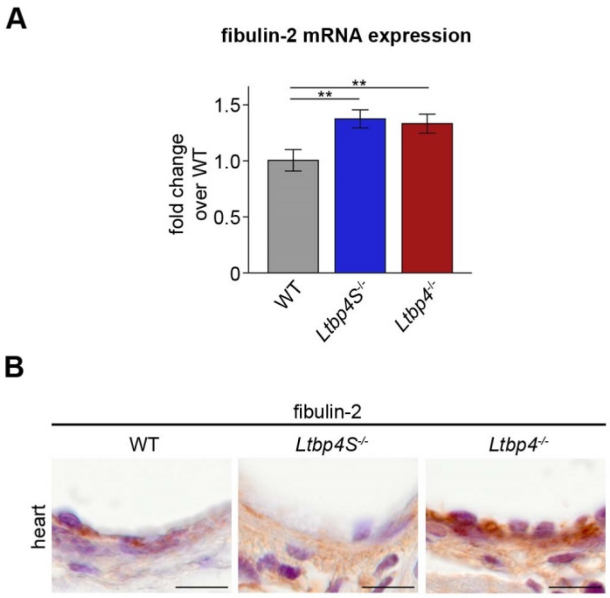

Supplementary material Fig. S7. Fibulin-2 is upregulated in heart of Ltbp-4 deficient mice.

(A) mRNA expression of fibulin-2 showed significant increase in hearts from *Ltbp4S*$^{-/-}$ and *Ltbp4*$^{-/-}$ mice compared to WT mice. Fibulin-2 mRNA expression of WT mice was set to 1 ($n \geq 5$; $^{**}p<0.01$). (B) Representative images showed an increase of fibulin-2 immunoreactivity especially in the vicinity of the endothelial lining of the vessel walls in hearts of *Ltbp4S*$^{-/-}$ and *Ltbp4*$^{-/-}$ mice compared to WT (Scale bar= 20 μm).

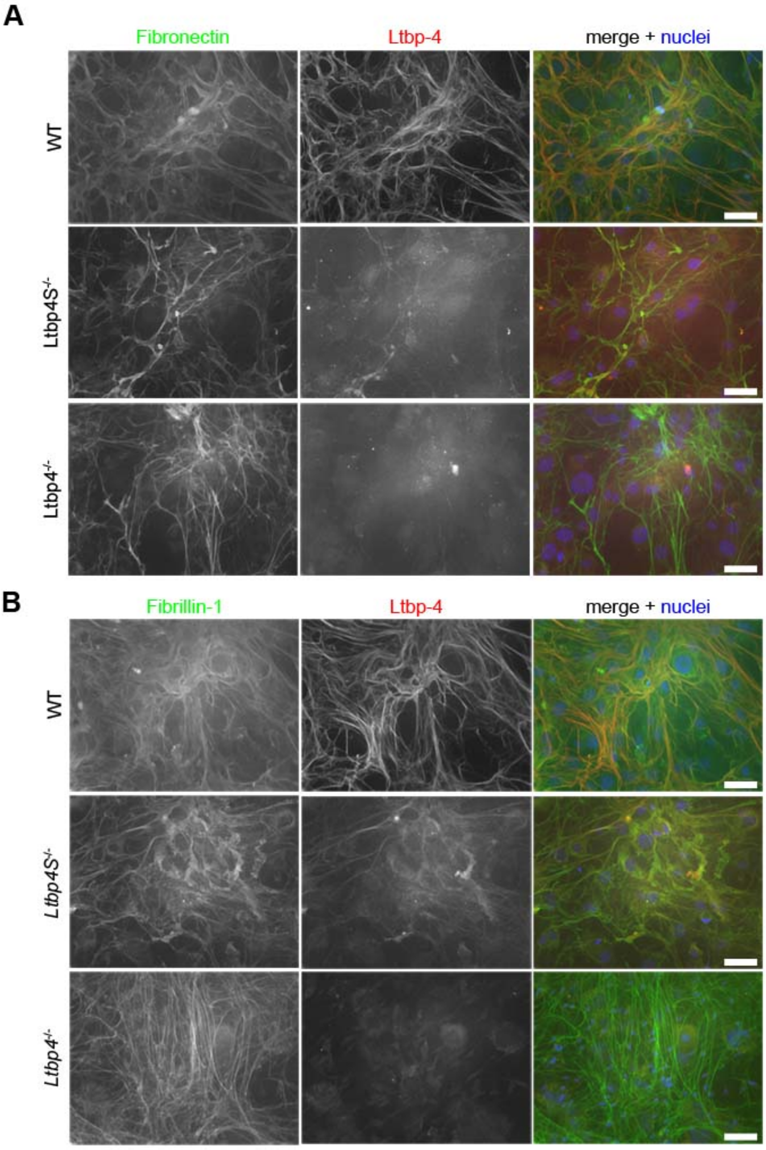

Supplementary material Fig. S8. Fibronectin and fibrillin-1 network assembly is normal in *Ltbp4S*$^{-/-}$ and *Ltbp4*$^{-/-}$ mice.

Immunofluorescence images of assembled fibronectin and fibrillin-1 networks by primary lung fibroblasts showed no differences between *Ltbp4S*$^{-/-}$ and *Ltbp4*$^{-/-}$ mice compared to WT mice. (Scale bar= 50 μm).

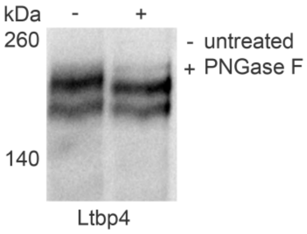

Supplementary material Fig. S9. Deglycosylation of Ltbp-4 isolated from WT lungs. Deglycosylation digests with PNGase F of WT lungs showed a shift towards lower molecular weight positions.

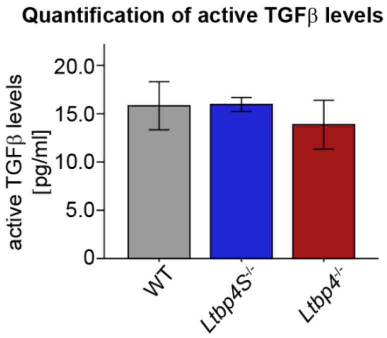

Supplementary material Fig. S10. Active TGFβ levels are comparable in *Ltbp4S*$^{-/-}$, *Ltbp4*$^{-/-}$ and WT mice.

Conditioned media from isolated lung fibroblasts were collected and assayed for active TGFβ levels. No differences between *Ltbp4S*$^{-/-}$ and *Ltbp4*$^{-/-}$ mice compared to WT mice were detected (n= 3; p= n.s.).

Supplementary material Table S1. Left-ventricular function is normal in *Ltbp4*$^{-/-}$ mice.

	**EF** [%]	**LVEDV** [µl]	**LVESV** [µl]	**SV** [µl]
**WT (n= 12)**	72.2 ±9.5	12.8 ±1.9	3.5 ±1.3	9.3 ±1.9
***Ltbp4*$^{-/-}$ (n= 8)**	76.1 ±1.1	11.5 ±0.2	2.8 ±0.1	8.8 ±0.3

The left ventricular end-diastolic volume (LVEDV), the left ventricular end-systolic volume (LVESV) and the stroke volume (SV) tended to be lower and the ejection fraction (EF) tended to be higher but did not reach statistical significance in *Ltbp4*$^{-/-}$ mice compared to WT mice as measured by transthoracic echocardiography (p= n.s.).

Supplementary material Table S2. Binding affinities for Ltbp-4L-2xStrep and Ltbp-4S-2xStrep interactions with rfibulin-4 and rfibulin-5.

**analyte / ligand**	**k_{on} *10^{-3} [$nM^{-1}s^{-1}$]**	**k_{off} *10^{-3} [s^{-1}]**	**K_d [nM]**
Ltbp-4L-2xStrep / rfibulin-5	2.2	5.3	2.4
Ltbp-4S-2xStrep / rfibulin-5	1.0	15.8	15.8
Ltbp-4L-2xStrep / rfibulin-4	1.3	4.1	3.1
Ltbp-4S-2xStrep / rfibulin-4	0.7	10.8	15.4

Surface plasmon resonance data measuring the binding affinity for immobilized recombinant full-length fibulin-4 and fibulin-5 (rfibulin; as ligand) and N-terminal Ltbp-4L (Ltbp-4L-2xStrep) and Ltbp-4S (Ltbp-4S-2xStrep) fragments flown over in solution (as analyte).

Supplementary material Table S3. Primers and probes.

5mL4_gen	5’-CTCTGGGTGTCGCTATTGGT-3’
3mL4_gen	5’-CAAGTCCATCCCCACACTCT-3’
betageo_gen	5’-GAAAGACCGCGAAGAGTTTG-3’
3C7WT	5’-GGCTCATGCTTGAATGTTTCAG-3’
3C7P3	5’-CCAATCTTGCTTCTTTGCTGAGC-3’
3C7TG	5’-ATCATGCAAGCTGGTGGCTG-3’
Ltbp4_SV<br>(Primerdesign, Rownhams, Southampton, UK)	Forward 5’-GGGACCCGGCTTCCGC-3’<br>Reverse 5’-GCAGCGATCAGGCTTCACA-3’<br>Probe 5’-CCTTCCTATGTCCCTTGATCTGTCACAACGtggaagg-3’
Ltbp4_LV<br>(Primerdesign, Rownhams, Southampton, UK)	Forward 5’-GTTGCTGCCTGTCACTGC-3’<br>Reverse 5’-GCACCCCTCTAGACCTGTG-3’<br>Probe 5’-CCTAGACCAGACACCTAAGAGTAGCCGCgtctagg-3’
Tropoelastin	Forward 5’-TTGCTGATCCTCTTGCTCAAC-3’<br>Reverse 5’-GCCCCTGGATAATAGACTCCAC-3’
Fibulin-2	Forward 5’-TGGTACCTGCACATATCTTCCGCA-3’<br>Reverse 5’- ACCACACCAGTGTAGGCATTGAGT-3’
Fibulin-4	Forward 5’-TGGTGCCTACAATGCCTTTC-3’<br>Reverse 5’-GGCGCTGACATTGTTGATTT-3’
Fibulin-5	Forward 5’-CCGATACCCTGGTGCCTATT-3’<br>Reverse 5’-GCACTGATAGGCCCTGTTTG-3’
Gapdh	Forward 5’-ATGTGTCCGTCGTGGATCTGA-3’<br>Reverse 5’-TGCCTGCTTCACCACCTTCT-3’

Supplementary material Table S4. Primary antibodies.

		Immuno-blot	Immuno-histochemistry	Immuno-fluorescence analysis
αSMA	spring bioscience, USA		1:100	
Ltbp-4	AF2885; R&D Systems, Germany	1:1000	1:100	1.2000
Fibulin-2	ab125256; abcam, UK		1:150	
Fibulin-4	kind gift from Takako Sasaki, Oita University, Japan	1:2000	1:400	1:2000
Fibulin-5	#3775; Epitomics, USA	1:5000	1:500	1:5000
PCNA	ab2426; abcam, UK		1:6000	
Strep-tag® fusion proteins	2-1507-001; IBA; Germany			1:2000

References

Sterner-Kock, A., Thorey, I. S., Koli, K., Wempe, F., Otte, J., Bangsow, T., Kuhlmeier, K., Kirchner, T., Jin, S., Keski-Oja, J. et al. (2002). Disruption of the gene encoding the latent transforming growth factor-beta binding protein 4 (LTBP-4) causes abnormal lung development, cardiomyopathy, and colorectal cancer. *Genes & Development* **16**, 2264-73.
